# Anaemia among women of reproductive age in selected sub-Saharan African countries: multivariate decomposition analyses of the demographic and health surveys data 2008–2018

**DOI:** 10.3389/fpubh.2023.1128214

**Published:** 2024-01-05

**Authors:** Mohammed Gazali Salifu, Frances Baaba Da-Costa Vroom, Chris Guure

**Affiliations:** ^1^Department of International Health, Bloomberg School of Public Health, The Johns Hopkins University, Baltimore, MD, United States; ^2^Department of Biostatistics, School of Public Health, College of Health Sciences, University of Ghana – Legon, Accra, Ghana; ^3^Department of Global Health and Population, Harvard T.H. Chan School of Public Health, Boston, MA, United States

**Keywords:** anaemia, women of reproductive age, Ghana, Sierra Leone, Benin, Mali, multivariate decomposition

## Abstract

**Objectives:**

The burden and highest regional prevalence of anaemia is reported in sub-Saharan Africa (SSA). The study evaluated changes in anaemia prevalence across the Demographic Health Surveys (DHS) periods in SSA and reported factors influencing observed changes in the trend.

**Method:**

The study was implemented by a two-stage cross-sectional stratified sampling approach. The study involved women of reproductive age (15–49 years) in sub-Saharan Africa countries (Ghana, Sierra Leone, Mali, and Benin) using two different periods of their demographic health surveys (DHS) data. The study adopted both descriptive and inferential statistical methods. The chi-square test was used to determine the existence of a statistically significant relationship between the outcome and predictor variables and test the observed changes in anaemia. Multivariable logistic regression analyses were conducted on each survey year and the pooled dataset for eligible study countries. Multivariate decomposition analysis was performed to explain how compositional changes and behavioural effects of women characteristics affected the changes in anaemia prevalence. The study reported frequencies, percentages and odds ratios along with their 95% confidence intervals (CI).

**Results:**

Ghana and Sierra Leone experienced 17.07% [95% CI: 14.76–19.37, *p* < 0.001] and 1% [95% CI: 1.0–2.9, *p* > 0.05] of anaemia decrease from period 1 to period 2, respectively, while Mali and Benin experienced 11% [95% CI: 9.14–12.90, *p* < 0.001] and 16.7% [95% CI: 14.99–18.5, *p* < 0.001] of increase in anaemia prevalence from period 1 to period 2, respectively. Behavioural effects explained the decrease in Ghana and the increase in Benin and Mali while endowments or compositional changes explained the decrease in Sierra Leone.

**Conclusion:**

Anaemia continues to pose a significant challenge in sub-Saharan Africa. Therefore, there is an imperative need to scale up the implementation of nutrition-related programmes and advocacies to ensure optimum changes in women nutrition-related behaviours.

## Introduction

Anaemia is considered a major public health condition with adverse health consequences. Anaemia is a medical condition resulting from inadequate red blood cells count or their oxygen-carrying capacity to meet the physiological needs of a human body (1), greatly varying with age, sex, altitude, smoking status, and pregnancy status (1).

Globally, 613.2 million women within the reproductive age (15–49 years) including 35.3 million non-pregnant women are affected by anaemia (2). South Asia and sub-Saharan Africa (SSA) regions have been identified with the lowest haemoglobin concentrations and highest anaemia prevalence over time (3). According to the 2016 Global Health Observatory Data Repository, 14 countries in the SSA, including Mali, Niger, Chad, Benin, Nigeria, Burkina Faso, Ghana, Togo, Cote D’ivoire, Guinea, Sierra Leone, Senegal, Gambia, and Guinea Bissau, have severe anaemia prevalence exceeding 40% among women of reproductive age (15–49 years). Countries among this region make up approximately half of 20 countries with the highest prevalence of anaemia worldwide (4). Anaemia global prevalence from 1995 to 2011 decreased from 33 to 29% in non-pregnant women (3). In addition, between 1993 and 2013, anaemia prevalence globally improved by 0.2–0.3% (5, 6).

Similarly, variation in anaemia trends among women of reproductive age (15–49 years) has been observed among countries in sub-Saharan Africa (SSA) with respect to their recent DHS reports. These reports include Benin (2012: 41.4%, 2017: 57.7%), Mali (2012: 51.4, 2018: 63.4%), Sierra Leone (2008: 45.2% 2013: 44.8%), and Senegal (2010: 54.3%, 2017: 54.1%) (7, 8). These persistent chronic anaemia prevalence rates within SSA regions are posing a greater challenge to achieving the global target of 50% reduction in anaemia prevalence in women of reproductive age by 2025 (9).

The factors influencing anaemia are often classified into two groups, namely, nutritional and non-nutritional based on their underlying causes (10). The most prevalent risk factors in low- and middle-income countries are nutritional deficiencies, infections, inflammations, and genetic haemoglobin disorders (11). The nutritional factors are mainly micronutrient deficiencies, including folate, vitamin B12, and vitamin A (11). The identified biological factors are chronic inflammation, parasitic infections (HIV and tuberculosis), and genetic conditions, such as thalassemia and sickle cell (12). Iron deficiency accounts for approximately 50% of all anaemia cases, indeed varying across countries (3, 13, 14). Sociodemographic factors are also identified to influence the occurrence of anaemia, including educational level of mothers, wealth index, educational level, age, and smoking status (15–19).

Several health, social, and economic effects of anaemia are identified (20, 21). Increased adverse pregnancy and child outcomes, such as maternal deaths, perinatal mortality, preterm delivery, and low birth weight, have been identified (22, 23). Furthermore, the morbidity linked with anaemia includes impaired work capacity, cognitive impairment, and increased susceptibility to infections (24, 25). Iron deficiency anaemia (IDA) contributes to over 100,000 maternal and 591,000 perinatal deaths annually as a result of low intake of absorbable dietary iron during adolescence and pregnancy (1). These mortality rates observed threaten the achievements of the Sustainable Development Goal (SDG) 3 targets, specifically the target of reducing the global maternal mortality ratio to less than 70 per 100,000 live births by 2030 (26).

Globally, considerable efforts have been made to increase anaemia awareness and advocacy on its adverse health effects and its developmental consequences on women and children. In the 65th World Health Assembly in 2012, action plan and global nutrition targets for maternal, infant, and child nutrition were approved. The commitment to reducing the prevalence of anaemia by 50% in women of reproductive age by 2025 based on the 2012 rate as a baseline measure was also approved (9). Currently, progress is off track to meet most of these world targets of nutrition, especially the anaemia target. However, countries, such as Burundi, Colombia, Kenya, Vanuatu, and Vietnam, are making good progress with reducing their population burden of anaemia (27).

There is an imperative need to reduce the high anaemia prevalence in the region and accelerate efforts towards achieving the global nutrition target of 50% reduction of anaemia in women of reproductive age by 2025. Therefore, finding strategic innovative measures to reduce anaemia burden and its adverse effects is imminent. This issue calls for a more extensive statistical analysis of nationally representative surveys conducted in the region to identify factors that have over the years contributed significantly more or less to the observed alarming anaemia prevalence within and among countries in the region. This study reports on the change in anaemia and its consistent predictors and factors contributing to changes in prevalence among women of reproductive age (15–49 years) in selected countries in the SSA region using the recent standard DHS. Findings of this study will provide further information to increase attention to improve investments in, and enhance acceleration of cost-effective interventions and policies towards addressing the high burden of anaemia in the region. Countries were included in this study based on the availability of DHS data and prevalence of anaemia.

### Conceptual framework

The conceptual framework shown in the diagram is delineated from various studies, which are reviewed to provide underpinning for this current study. The low number of red blood cells or their inadequate oxygen-carrying capacity to meet the minimum physiological needs of a human body biologically causes anaemia. However, factors influencing anaemia are multifaceted and functionally linked, synergistically acting to manifest their effects.


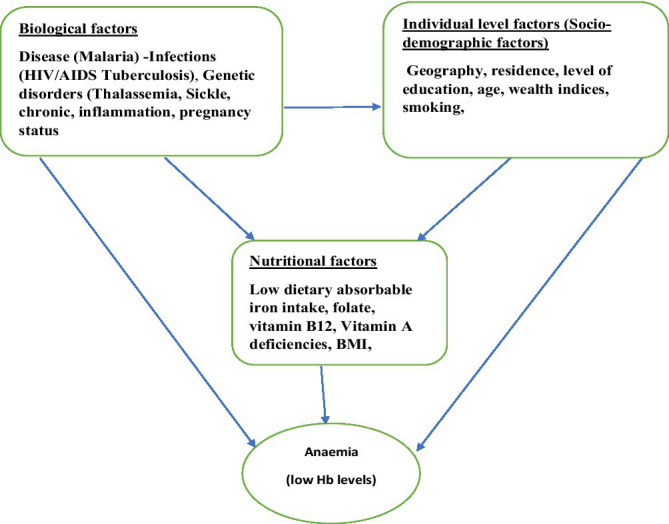


The determinants of anaemia are often classified into two, namely, nutritional and non-nutritional, based on its underlying causes according to the World Health Organization. The most prevalent risk factors in low- and middle-income countries are nutritional deficiencies, infections, inflammations, and genetic haemoglobin disorders (11). The nutritional factors are mainly micronutrient deficiencies, including folate, vitamin B12, and vitamin A (11). Iron deficiency alone accounts for approximately 50% of all anaemia cases, but it may vary by country and region and account for less than the 50% reported (14). The biological factors identified are chronic inflammation, parasitic infections (HIV and tuberculosis), and genetic conditions, such as thalassemia and sickle cell (12). Sociodemographic factors also identified to influence the occurrence of anaemia, such as educational level of mothers, wealth index, educational level, age, and smoking status (15–19).

## Methodology

### Study design

This study uses data from the standard Demographic Health Surveys conducted in sub-Saharan African (SSA) countries. Demographic Health Surveys employ retrospective cross-sectional survey designs. These surveys are periodically conducted in developing countries, including SSA, with support from International Classification of Functioning, Disability and Health (ICF).

### Study area

According to The World Bank Group (2020), 48 countries in Africa constitute sub-Saharan Africa (SSA). However, this current study selected four SSA countries (Ghana, Benin, Mali, and Sierra Leone). These countries were chosen based on their anaemia prevalence among women of reproductive age. First, countries that included the indicator “women with any anaemia” in their two most recent standard DHS were delineated. Second, countries were selected based on an interval difference of 5–7 years between their two most recent standard DHS.

### Sample design

In Ghana, Benin, Mali, and Sierra Leone, eligible women within the age group of 15–49 years who were visitors or occupants of a household prior to the night of the survey participated. DHS samples interviewed are representative at the national and regional levels. Two-stage stratified sampling design was employed by DHS. The first stage involves drawing enumeration areas from the most recent census files or documents, and the second stage draws a sample of households from enumeration areas (7). Information is solicited, and tests including malaria and anaemia and blood samples for HIV are conducted on participants upon their individual approvals. Women within the age group of 15–49 years, who voluntarily consented, provided blood specimen for anaemia testing. Microcuvette was used to collect blood samples drawn from a drop of blood taken from a finger prick. A battery-operated portable HemoCue analyser was used for onsite haemoglobin analysis. The study grouped the survey years into periods. The first survey year was clustered as period one, the second survey year was clustered as period two, and the combination of first and second survey years was clustered as pooled data.

### Study population

The population of this study included all women within the age of 15–49 years who were selected and granted their consent for anaemia testing. All participants included had complete information/data.

### Patient and public involvement

Neither patients nor the public were involved in the design, planning, conducting, or reporting of this study.

### Sample size

A total of 91,814 samples for all survey years for the four countries were obtained from DHS for specifically answering the research questions and achieving the specific objectives of the study. Since this study focuses on anaemia among women within the ages of 15–49 years, only those who were selected and granted their consent for anaemia testing with complete information/data were eligible to participate in the study. After excluding all non-eligible participants, the overall data set for the study summed up to 45,328 (refer to Table 1 for each country and the specific sample size utilized).

**Table 1:** Sample sizes of countries eligible for the study.

	Periods groupings				
Country	DHS year	Period 1	DHS year	Period 2	Pooled data
Ghana	2008	3,488	2014	7,704	11,192
Benin	2011–12	5,049	2017–18	8,011	13,060
Mali	2012–13	5,160	2018	5,090	10,250
Sierra Leone	2008	3,122	2013	7,704	10,826
Total		16,819		28,509	45,328

## Data variables

### Dependent variable

In the DHS, anaemia is defined on the basis of WHO cut-off points of haemoglobin published in 1968 by a WHO study group on nutritional anaemia (1), as shown in Table 2.

**Table 2:** Haemoglobin classification for non-pregnant women aged 15–49 years.

Anaemia classification	Non-pregnant (Hb levels (g/L))	Pregnant women (Hb levels (g/L))
Non-anaemia	120 or more	110 or higher
Mild	110–119	100–109
Moderate	80–109	70–99
Severe	Less than 80	Less than 70

However, this present study re-categorized the occurrence of anaemia as a binary outcome due to the statistical techniques employed. Thus, non-pregnant women with Hb levels of 120 g/L or more were coded “No” implying non-anaemic and those with Hb levels of 119 g/L or less were coded “Yes” also implying anaemic. Pregnant women with Hb levels of 110 g/L or more were coded “No” implying non-anaemic and those with Hb levels of 100 g/L or less were coded “Yes” also implying anaemic (1).

### Independent variables

The study considered several independent variables, including sociodemographic characteristics of participants such as age, religion, highest educational level, wealth index, residence, marital status, working status, smoking status, coverage of health insurance status, listening to radio, watching television, and reading newspaper/magazine; and biological factors such as number of children ever born, breastfeeding status, pregnancy status, and usage of hormonal contraceptives. Specifically, the term folkloric or traditional methods of contraceptives are often classified in the DHS as periodic abstinence (rhythm, calendar method), withdrawal (coitus interrupts), or country-specific traditional methods of proven effectiveness, and folk methods include locally described methods and/or spiritual methods of unproven effectiveness, such as herbs, amulets, and gris-gris (Table 3).

**Table 3:** Categorizations of various independent variables included in the analyses.

Variables	Categorizations for analyses
Mother’s age (years)	15–19 20–24 25–29 30–34 35–39 40–44 45–49
Marital status	Single Married Cohabiting Separated/widowed/divorced
Highest level of education attained	No education Primary Secondary Higher
Place of residence	Rural Urban
Wealth index	Poorest Poorer Middle Richer Richest
Religion	Christians Muslim Traditionalists Others
Currently working	No Yes
Currently pregnant	No or unsure Yes
Number of children ever born	0 1–3 4+
Currently breastfeeding	No Yes
Anaemia level	Severe Moderate Mild Non anaemic
Smokes cigarette	No Yes
Covered by health insurance	No Yes
Current method of contraceptives	No method Folkloric method Traditional method Modern method
Reads newspaper	Yes No
Watches television	Yes No
Listens to radio	Yes No

### Statistical analysis

The analysis was performed on each survey period for each of the four countries and their pooled data set. The study adopted both descriptive and inferential statistical methods. Frequencies (sociodemographic factors and biological factors), percentages (sociodemographic factors and biological factors), and odds ratios (sociodemographic factors and biological factors) were reported at 5% significance level. Cross-tabulation was used to determine the existence of a relationship between the outcome variable and the predictor variable for each survey year. The chi-square test was carried out to test the anaemia trend observed across the survey years in each country. Multivariable logistic regression analyses were carried out on each survey period for the four countries. Variables included at the multivariable level were identified from the chi-square tests to be statistically significant. Some important variables identified from the literature were also included at the multivariable level, irrespective of their *p*-values. This study used a standard multivariate decomposition analysis technique (two-component), which is mostly used to analyse differences between two groups or time points. This technique has been used in several studies in public health to identify and quantify components of change over time and also identify factors associated with the change. This analytical approach decomposes the differences in two time points into two components, in which case, the first component is the change that is a result of the variation in the survey population structure, and the second component is the result of the change in public health or the behaviour of the survey population. This analysis was carried out to ascertain the effect of the difference between the unexplained variables and the difference between the explained variables on the dependent variable. This statistical approach was executed using Stata Statistical Software version 16, College Station, TX: StataCorp LP (Stata) (28). The complex survey approach, which takes into consideration stratification and weights, was used to estimate means, proportions, coefficients, and confidence intervals.

### Multivariate decomposition

Multivariate decomposition, as propounded by Oaxaca and Blinder in 1973, states that the difference in two groups could be explained by the composition of the endowment or the behavioural effect of these characteristics (coefficients). This decomposition utilized estimates from the logit models. The endowment looks at how the change in the distribution affects the change in outcome over the two points in time. The coefficient looks at the actual effect of change in the outcome variable (29).

## Results

### Changes in the distribution of women’s characteristics from periods 1 to 2 in countries (Ghana, Mali, Sierra Leone, and Benin)

In Ghana, the distribution of women aged 15–19 years decreased appreciably by 3.77 percentage points [890 (24.37%)–832 (20.6%)] from period 1 to period 2, while the distribution of women aged 30–34 years increased by 3.07% [401 (11.79%)–493 (14.86%)]. The distribution of married women declined by 2.36% [1,353 (38.32)–1,294 (35.96)] from period 1 to period 2. The distribution of women with the primary level of education experienced a decrease by 3.26 percentage points [959 (25.84)–886 (22.58)] from period 1 to period 2, and the distribution of those with higher level of education increased by 2.5% [160 (4.66)–237 (7.16)]. Women with zero parity distribution decreased by 3.07% [1,403 (39.57)–1,349 (36.5)], while women with 1–3 parity increased by 2.95% [1,337 (38.83)–1,434 (41.78)] from period 1 to period 2 in Ghana (Table 4).

**Table 4:** Weighted frequency and percentage distribution of women’s characteristics, period 1, period 2, and pooled data of Ghana, Mali Benin, and Sierra Leone DHS.

Background characteristics	% Change in characteristics Ghana	% change in characteristics Mali	% change in characteristics Benin	% change in characteristics Sierra Leone
Mother’s age (years) 15–19 20–24 25–29 30–34 35–39 40–44 45–49	−3.77 [24.37, 20.6] −0.88 [19.49, 18.61] −0.18 [17.31, 17.13] 3.07 [11.79, 14.86] 0.32 [12.07, 12.39] 1.02 [8.06, 9.08] 0.39 [6.92, 7.31]	1.47 [17.71, 19.18] −0.07 [17.84, 17.77] −0.79 [19.95, 19.16] −0.01 [15.95, 15.94] 1.25 [11.86, 13.11] −1 [9.57, 8.57] −0.85 [7.13, 6.28]	3.63 [17.1, 20.73] 1.82 [16.49, 18.31] −1.22 [19.41, 18.19] −2.59 [16.88, 14.29] −1.32 [12.92, 11.58] −1.82 [10.51, 8.69] 1.53 [6.69, 8.22]	6.1 [15.29, 21.39] 0.62 [15.83, 16.45] −5.32 [22.65, 17.33] 0.79 [13.61, 14.4] −2.27 [16.48, 14.21] −0.94 [9.06, 8.12] 1.02 [7.08, 8.104]
Marital status Single Married Cohabiting Separated/widowed/divorced	−0.49 [38.6, 38.11] −2.36 [38.32, 35.96] 1.12 [14.14, 15.26] 1.71 [8.95, 10.66]	1.75 [13.52, 15.27] −1.55 [82.83, 81.28] −1.2 [1.79, 0.60] 0.99 [1.86, 2.85]	0.82 [22.98, 23.8] 2 [53.62, 55.62] −3.20 [18.03, 14.83] 0.37 [5.38, 5.75]	10.45 [16.51, 26.96] −3.9 [67.75, 63.85] −6.82 [9.49, 2.68] 0.27 [6.25, 6.51]
Highest level of education attained Primary Secondary Higher	−3.26 [25.84, 22.58] 0.75 [69.5, 70.25] 2.5 [14.66, 7.16]	−8.42 [75.26, 66.84] 3.51 [9.642, 13.15] 4.23 [13.9, 18.13] 0.68 [1.196, 1.881]	−5.4 [60.91, 55.51] 2.9 [16.53, 19.43] 2.72 [20.52, 23.24] −0.22 [2.042, 1.817]	−12.1 [68.42, 56.32] 1.43 [12.4, 13.83] 9.41 [17.38, 26.79] 1.26 [1.8, 3.06]
Place of residence Rural Urban	−5.3 [45.64, 40.34] 5.3 [54.36, 59.66]	1.75 [23.81, 25.56] −1.75 [76.19, 74.44]	−3.62 [45.19, 41.57] 3.62 [54.81, 58.43]	2.1 [33.87, 35.97] −2.1 [66.13, 64.03]
Wealth index Poorest Poorer Middle Richer Richest	1.74 [8.09, 9.83] −1.53 [17.09, 15.56] 0.87 [21.33, 22.2] −0.95 [21.33, 22.2] −0.14 [27.34, 27.2]	−2.88 [20.01, 17.13] 2.27 [18.17, 20.44] −0.28 [19.43, 19.15] 1.24 [19.82, 21.06] −0.35 [22.57, 22.22]	−0.66 [16.74, 16.08] 0.26 [17.81, 18.07] 1.28 [19.46, 20.74] 1.6 [20.62, 22.22] −2.47 [25.36, 22.89]	−0.38 [18.4, 18.02] −0.91 [19.94, 19.03] −0.78 [19.95, 19.17] −1.21 [20.92, 19.71] 3.29 [20.79, 24.08]
Religion Christians Muslim Traditionalists Others	1.39 [85.2, 86.59] 0.14 [10.63, 10.77] −0.76 [1.87, 1.11] −0.78 [2.31, 1.53]	−1.9 [4.56, 2.66] 1.63 [92.14, 93.77] 0.28 [3.30, 3.58]	−2.6 [57.62, 55.02] 7.83 [21.66, 29.49] −4.27 [13.41, 9.138] −0.95 [7.304, 6.352]	−1.94 [23.16, 21.22] 2.56 [75.97, 78.53] −0.21 [0.06, 0.27] −0.62 [0.81, 0.22]
Currently working No Yes	5.2 [29.0, 34.2] −5.2 [71.0, 65.8]	−10.62 [55.51, 44.89] 10.62 [44.49, 55.11]	−17.9 [42.17, 24.27] 17.9 [57.83, 75.73]	6.11 [26.21, 32.32] −6.11 [73.79, 67.68]
Currently pregnant No or unsure Yes	0.04 [93.07, 93.11] −0.04 [6.93, 6.89]	0.29 [88.21, 88.5] −0.29 [11.79, 11.5]	−1.27 [90.4, 89.13] 1.27 [9.60, 10.87]	3.47 [88.02, 91.49] −3.47 [11.98, 8.51]
Number of children ever born 0 1–3 4+	−3.07 [39.57, 36.5] 2.95 [38.83, 41.78] 0.11 [21.61, 21.72]	2.24 [17.34, 19.58] −2.48 [38.93, 36.45] 0.24 [43.73, 43.97]	1.69 [23.72, 25.41] −2.87 [39.76, 36.89] 1.18 [36.52, 37.7]	7.17 [16.77, 23.94] −6.47 [44.16, 37.69] −0.71 [39.07, 38.36]
Currently breastfeeding No Yes	−1.66 [81.97, 80.31] 1.66 [18.03, 19.69]	−0.21 [63.75, 63.54] 0.21 [36.25, 36.46]	−2.46 [73.39, 70.93] 2.46 [26.61, 29.07]	4.3 [70.55, 74.85] −4.3 [29.45, 25.15]
Anaemia level Severe Moderate Mild Non anaemic	−1.78 [2.181, 0.41] −8.21 [17.37, 9.16] −7.23 [38.91, 31.68] 16.86 [41.54, 58.4]	2.98 [1.34, 4.32] 20.94 [13.29, 34.23] −11.92 [36.79, 24.87] −12 [48.59, 36.59]	1.34 [0.53, 1.87] 21.78 [8.46, 30.24] −6.78 [32.4, 25.62] −16.34 [58.61, 42.27]	−0.07 [0.65, 0.58] −1 [10.8, 9.80] 0.58 [33.74, 34.32] 0.49 [54.81, 55.3]
Smokes cigarette No Yes	1.07 [99.83, 99.9] −0.07 [0.17, 0.10]	−0.59 [99.91, 99.32] 0.59 [0.09, 0.68]	−1.33 [99.89, 98.56] 1.33 [0.11, 1.44]	1.36 [93.97, 95.33] −1.36 [6.03, 4.67]
Covered by health insurance No Yes	−20.53 [58.02, 37.49] 20.53 [41.98, 62.51]	−1.99 [96.82, 94.83] 1.99 [3.179, 5.17]	0.77 [98.17, 98.94] −0.77 [1.83, 1.06]	0.86 [98.2, 99.06] −0.86
Current method of contraceptives No method Folkloric method Traditional method Modern method	−2.27 [78.46, 76.19] −0.42 [0.66, 0.24] −1.34 [6.37, 0.24] 4.04 [14.50, 18.54]	−6.31 [89.72, 83.41] 0.13 [0.36, 0.49] 0.25 [0.07, 0.32] 5.94 [9.85, 15.79]	−0.77 [85.65, 84.88] −1.04 [4.03, 2.99] 3.32 [8.80, 12.12]	−11.3 [89.73, 78.43] −0.46 [1.16, 0.70] −0.3 [0.70, 0.40] 12.07 [8.41, 20.48]
Reads newspaper Yes No	−7.28 [29.29, 22.29] 7.28 [70.71, 77.99]	−2.12 [9.49, 7.37] 2.12 [90.51, 92.63]	−6.06 [14.99, 8.93] 6.06 [85.01, 91.07]	0.21 [10.19, 10.4] −0.21 [89.81, 89.6]
Watches television Yes No	9.79 [71.91, 81.70] −9.79 [28.09, 18.30]	14.53 [49.73, 64.26] −14.53 [50.27, 35.74]	−10.73 [49.29, 38.56] 10.73 [50.71, 61.44]	5.68 [14.8, 20.48] −5.68 [85.2, 79.52]
Listens to radio Yes No	−3.05 [89.48, 86.43] 3.05 [10.52, 13.57]	2.08 [68.69, 70.77] −2.08 [31.31, 29.23]	−7.51 [64.67, 57.16] 7.51 [35.33, 42.84]	6.63 [56.16, 62.79] −6.63 [43.84, 37.21]

Distributional changes regarding women characteristics were also found in Mali; women with no formal education reduced greatly by 8.42% [3,825 (75.26)–3,458 (66.84)] while women with primary and secondary education increased marginally by 3.51% [515 (9.642)–647 (13.15)] and 4.23% [751 (13.9)–895 (18.13)] from period 1 to period 2. The distribution of women working was also increased largely by 10.62% from period 1 to period 2. Certain changes in women distribution regarding biological factors were also observed in Mali. Furthermore, the number of women using modern methods of contraceptives was improved by 5.94% [530 (9.85) –714 (15.79)] (Table 4).

In addition, the distribution of women with no education reduced by 5.4% [3,222 (60.91)–4,437 (55.51)] while those with primary and secondary levels of education improved marginally by 2.9% [789 (16.53)–1,561 (19.43)] and 2.72% [964 (20.52)–1852 (23.24)] from period 1 to period 2 in Benin; likewise, the distribution of rural women was declined by 3.62% [2,083 (45.19)–3,485 (41.57)] from period 1 to period 2. Appreciable changes were also observed in women characteristics such as women’s age, breastfeeding status, and methods of contraceptive usage from period 1 to period 2 (Table 4).

Women distribution in Sierra Leone also observed larger changes from period 1 to period 2. Changes were mostly observed with women characteristics, such as women’s age, marital status, educational level, and working status. Regarding women’s age, women aged 15–19 years increased in number by 6.1% [514 (15.29)–1751 (21.39)] while those aged 25–29 years and 35–39 years reduced by 5.32% [677 (22.65)–1,270 (17.33)] and 2.27% [484 (16.48)–1,106 (14.21)] from period 1 to period 2 (Table 4).

### Anaemia prevalence and trends among women of reproductive age in Ghana, Mali, Sierra Leone, and Benin

**Figure 1:**
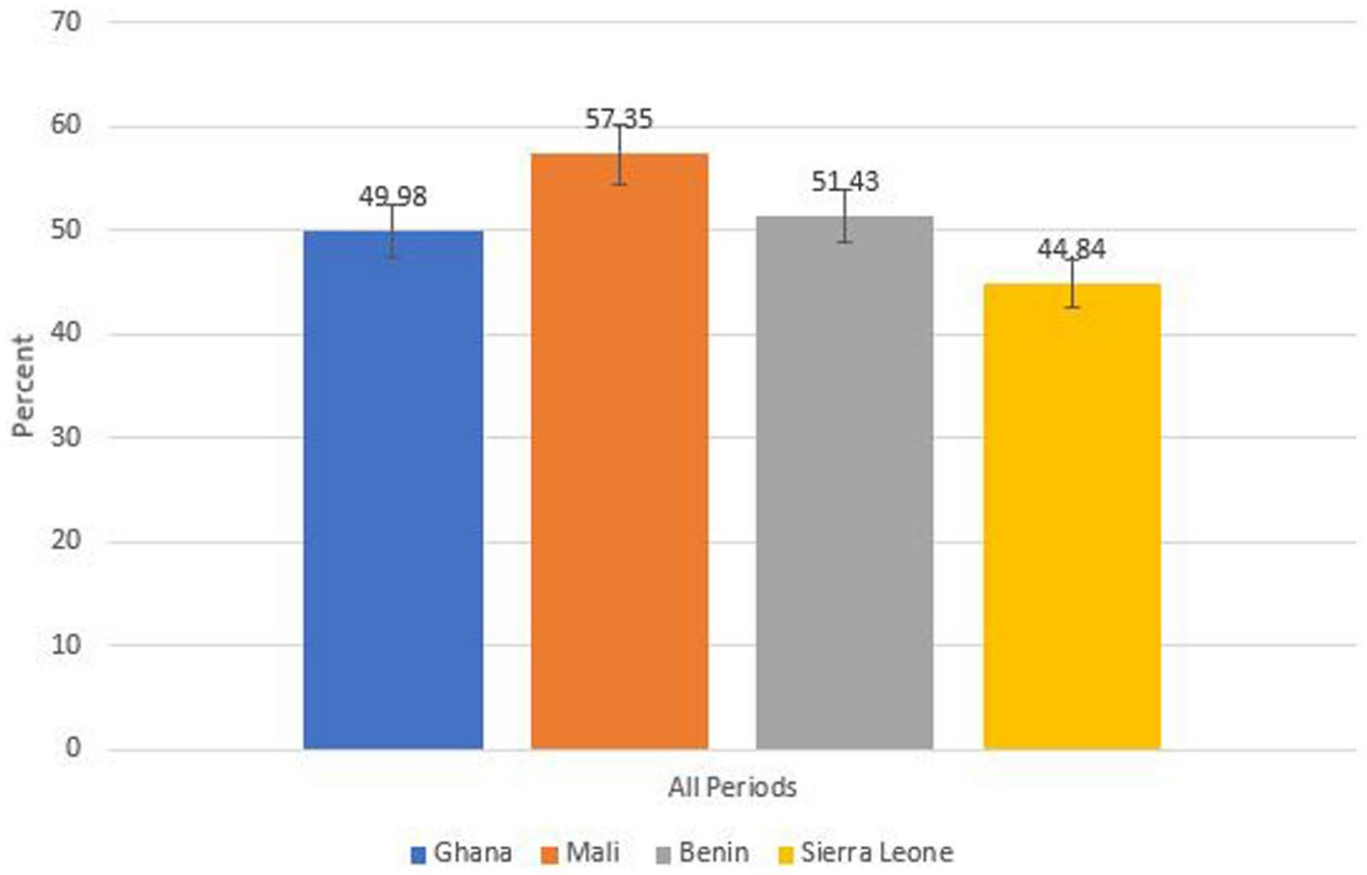
Anaemia prevalence among women of reproductive age in each country for all survey periods combined.

The prevalence of anaemia among women of reproductive age for all their survey periods for each country is shown in Figure 1. Mali had the highest prevalence, 57% [95% CI: 55.88–8.80]. The prevalence of anaemia in Benin was also observed to be higher, 51.43% [95% CI: 50.17–52.70]. In addition, the anaemia prevalence in Ghana for all the survey periods combined was 49.98% [95% CI: 48.34–51.62]. The least anaemia prevalence for all survey periods combined was observed in Sierra Leone, 44.84% [95% CI: 42.97–46.72].

**Figure 2:**
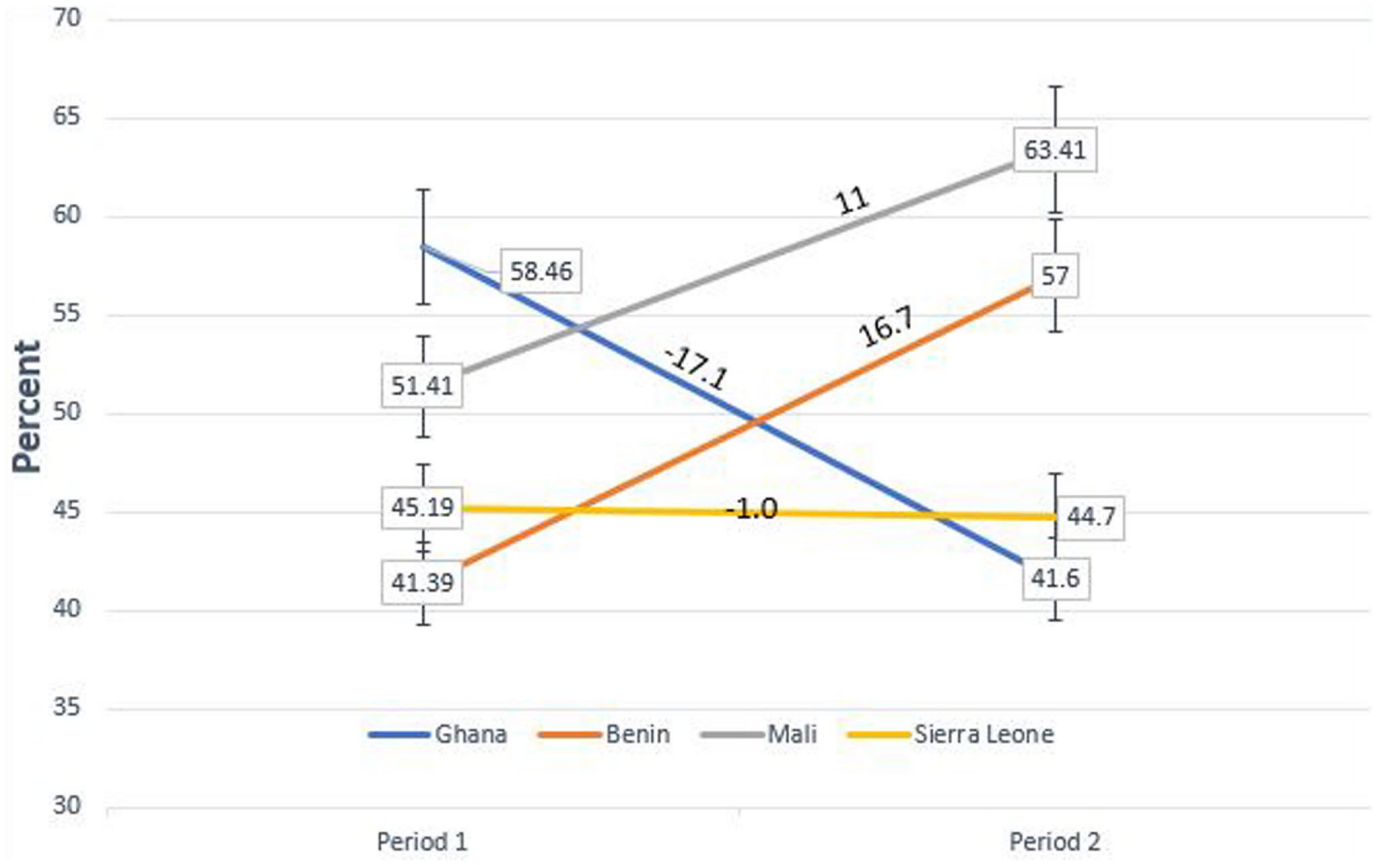
Trends of anaemia prevalence among women of reproductive age across all survey periods.

The trend and prevalence of anaemia among women of reproductive age in all the four countries are shown in Figure 2. Ghana experienced a large decrease in trend from 58.46% in period 1 to 41.6% in period 2, representing 17.07% reduction [95% CI: 14.76–19.37, *p* < 0.001]. Sierra Leone experienced a small decrease from 45.19% in period 1 to 44.7% period 2, representing 1% reduction [95% CI: 1.0–2.9, *p* > 0.05]. However, Mali experienced a large trend increase in anaemia prevalence from 51.41% in period 1 to 63.41% in period 2, representing 11% increase [95% CI: 9.14–12.90 p < 0.001]. Similarly, Benin also had a larger increase in anaemia prevalence from 41.39% in period 1 to 57% in period 2, representing 16.7% increase [95% CI: 14.99–18.5 *p* < 0.001].

### Changes in the distribution of anaemia prevalence across women’s characteristics among the four sub-Saharan African countries

In Ghana (Supplementary Table A), most of the changes were observed in women characteristics, such as age of women, marital status, educational level, residence, wealth index, working status, and coverage with health insurance. Single women anaemia prevalence dropped marginally by 5.35% from period 1 to period 2. Anaemia prevalence decreased by 3.85% from period 1 to period 2 among women with primary level of education. Furthermore, anaemia prevalence increased largely by 7.81% among women in urban areas.

Changes in women characteristics regarding anaemia prevalence observed in Mali included women’s age, levels of education, wealth index, and women’s working status. Women with no education anaemia prevalence decreased by 8.04% from period 1 and period 2; likewise, women in the poorest wealth category anaemia prevalence reduced by 3.82% from period 1 to period 2 (Supplementary Table B). In addition, women not using any method of contraceptive anaemia prevalence reduced by 5.58%.

Distributional changes in anaemia prevalence were also observed across women characteristics, such as women’s age, marital status, highest education, residence, religion, and working status in Benin from period 1 to period 2. In addition, the largest changes were observed with women cohabitating, women with secondary level of education, and women not currently working. Women working anaemia prevalence reduced largely by 15.97% from period 1 to period 2; likewise, women with secondary level of education anaemia prevalence also declined by 27.6% from period 1 to period 2 (Supplementary Table C).

Larger changes in anaemia prevalence distribution were observed in Sierra Leone. In addition, single women anaemia prevalence increased by 7.13% while women cohabitating anaemia prevalence dropped by 7.61% from period 1 to period 2. Moreover, anaemia prevalence of women with no education reduced by 7.61%. Furthermore, women who have 1–3 parity anaemia prevalence reduced by 7.51% from period 1 to period 2. In addition, women not using any method of contraceptive anaemia prevalence reduced by 7.51% while those using modern contraceptive anaemia prevalence increased greatly by 9.18% from period 1 to period 2 (Supplementary Table D).

### Period 1, period 2, and pooled data of multivariable logistic regression analysis in each country

#### Ghana

In period 1, women aged 25–29 years had 37% [aOR: 0.63 95% CI: 0.48–0.83, *p* < 0.01] reduced odds of developing anaemia compared with women aged 15–19 years. Women aged 30–34 years and 35–39 years also had 33% [aOR: 0.67 95% CI: 0.49–0.91, *p* < 0.01] and 28% [aOR: 0.72 95% CI: 0.52–0.99, *p* < 0.05] reduced odds of developing anaemia compared with women aged 15–19 years in Ghana. In addition, women with higher education had 47% [aOR: 0.53 95% CI: 0.34–0.84, *p* < 0.01] reduced odds of developing anaemia compared with non-educated women. Pregnant women had 1.64% [95% CI: 1.20–2.24, *p* < 0.01] increased odds of developing anaemia compared with non-pregnant women. In addition, women using the folkloric method of contraceptives had 2.97% [95% CI: 1.07–8.28, *p* < 0.05] more odds of anaemia than women not using any method of contraceptives. Moreover, with reference to period 2, women aged 35–39 years and 45–49 years had 28% [aOR:0.72 95% CI:0.47–1.09, *p* < 0.05] and 47% [aOR:0.63 95% CI: 0.37–1.03, *p* < 0.05] reduced odds of anaemia compared with women aged 15–19 years in Ghana. With the analysis of pooled datasets, women aged 25–29 years and 30–34 years had 32% [aOR: 0.68 95% CI: 0.54–0.85, *p* < 0.01] and 36% [aOR: 0.66 95% CI: 0.51–0.85, *p* < 0.01] reduced odds of developing anaemia compared with women aged 15–19 years in Ghana. In addition, women with secondary level of education had 16% [aOR: 0.84 95% CI: 0.74–0.96, *p* < 0.05] reduced odds; likewise, women with higher level of education had 44% [aOR: 0.56 95% CI: 0.42–0.76, *p* < 0.001] reduced odds of developing anaemia than those with primary level of education. Moreover, women within the poorer wealth index had 26% [aOR: 1.26 95% CI: 1.02–1.55, *p* < 0.05] increased odds of developing anaemia compared with women within the poorest wealth index. Women practicing other religions had 1.57% [95% CI: 1.09–2.25, *p* < 0.05] increased odds of developing anaemia against Christian women. In addition, pregnant women in Ghana had 1.30% [95% CI: 1.04–1.64, *p* < 0.05] increased odds of anaemia than non-pregnant women. Furthermore, women using modern contraceptives had 21% [aOR: 0.79 95% CI: 0.67–0.93, *p* < 0.01] reduced odds of developing anaemia compared with non-contraceptive users (Table 5).

**Table 5:** Factors influencing anaemia among women of reproductive age, period 1, period 2, and pooled data of Ghana and Sierra Leone DHS.

	Ghana			Sierra Leone		
Variables	Period 1	Period 2	Pooled data	Period 1	Period 2	Pooled data
	aOR (95% CI)	aOR (95%CI)	aOR (95%CI)	aOR (95% CI)	aOR (95%CI)	aOR (95%CI)
Age category						
15–19	1 [reference]	1 [reference]	1 [reference]	1 [reference]	1 [reference]	1 [reference]
20–24	0.79 [0.61–1.01]	1.11 [0.85–1.45]	0.91 [0.76–1.09]	0.78 [0.55–1.11]	0.71 [0.58–0.86]**	0.72 [0.61–0.86]***
25–29	0.63 [0.48–0.83]**	0.79 [0.54–1.14]	0.68 [0.54–0.85]**	0.94 [0.68–1.29]	0.64 [0.51–0.80]*	0.71 [0.59–0.85]***
30–34	0.67 [0.49–0.91] *	0.82 [0.55–1.23]	0.66 [0.51–0.85]**	0.88 [0.61–1.27]	0.57 [0.44–0.73]**	0.65 [0.54–0.81]***
35–39	0.72 [0.52–0.99]*	0.72 [0.47–1.09]*	0.65 [0.50–0.86]**	0.94 [0.65–1.35]	0.56 [0.43–0.74]**	0.67 [0.54–0.84]***
40–44	0.74 [0.51–1.07]	0.83 [0.53–1.30]	0.68 [0.51–0.91]*	0.76 [0.49–1.17]	0.48 [0.35–0.66]**	0.57 [0.45–0.74]***
45–49	0.66 [0.44–1.00]	0.63 [0.37–1.03]*	0.57 [0.42–0.79]**	0.74 [0.46–1.19]	0.53 [0.39–0.72]*	0.62 [0.48–0.80]***
Marital status						
Single		1 [reference]	1 [reference]	1 [reference]	1 [reference]	
Married		1.11 [0.82–1.52]	1.14 [0.92–1.42]	0.90 [0.68–1.19]	0.99 [0.85–1.16]	
Cohabiting		1.11 [0.82–1.50]	0.99 [0.80–1.24]	0.99 [0.75–1.34]	0.89 [0.73–1.07]	
Separate/widowed/divorced		1.38 [0.95–2.01]	1.22 [0.94–1.60]	0.64 [0.35–1.16]	0.74 [0.43–1.25]	
Highest level of education						
No education				1 [reference]	1 [reference]	1 [reference]
Primary	1 [reference]	1 [reference]	1 [reference]	0.90 [0.68–1.19]	1.01 [0.88–1.14]	0.99 [0.87–1.14]
Secondary	0.84 [0.69–1.02]	0.92 [0.75–1.12]	0.84 [0.74–0.96]*	0.99 [0.75–1.34]	0.84 [0.73–0.98]*	0.95 [0.81–1.11]
Higher	0.53 [0.34–0.84] **	0.71 (0.48–1.05)	0.56 [0.42–0.76]***	0.64 [0.35, 1.16]	0.44 [0.27–0.93]**	0.72 [0.45–1.14]
Place of residence						
Urban	1 [reference]		1 [reference]		1 [reference]	1 [reference]
Rural	1.17 [0.93–1.48]		0.98 [0.84–1.16]		1.24 [0.94–1.63]	1.07 [0.86–1.36]
Wealth index						
Poorest	1 [reference]	1 [reference]	1 [reference]	1 [reference]	1 [reference]	1 [reference]
Poorer	1.11 [0.84–1.48]	1.32 [0.98–1.79]	1.26 [1.02–1.55]*	1.16 [0.86–1.56]	0.94 [0.77–1.13]	0.99 [0.84–1.16]
Mild	1.03 [0.76–1.42]	1.23 [0.93–1.65]	1.14 [0.92–1.42]	1.04 [0.79–1.37]	0.93 [0.77–1.12]	0.96 [0.82–1.12]
Richer	1.21 [0.87–1.68]	0.88 [0.66–1.18]	1.03 [0.81–1.30]	0.96 [0.69–1.32]	0.95 [0.74–1.21]	0.92 [0.76–1.12]
Richest	1.12 [0.78–1.62]	0.98 [0.72–1.34]	1.01 [0.78–1.32]	1.08 [0.78–1.49]	0.71 [0.49–1.03]	0.76 [0.57–1.01]
Religion						
Christians	1 [reference]		1 [reference]	1 [reference]	1 [reference]	1 [reference]
Moslems	1.08 [0.84–1.39]		0.97 [0.82–1.15]	1.33 [1.14–1.55]***	1.33 [1.14–1.55]***	1.22 [1.08–1.37]
Traditionalists	0.75 [0.47–1.21]		0.98 [0.64–1.50]	#	#	3.07 [0.32–29.26]
Others	1.60 [0.96–2.66]		1.57 [1.09–2.25]*	1.34 [0.46–3.88]	1.34 [0.46–3.88]	0.84 [0.45–1.57]
Currently working						
No		1 [reference]	1 [reference]			1 [reference]
Yes		0.84 [0.67–1.05]	0.93 [0.80–1.09]			1.52 [1.27–1.82]***
Current pregnancy						
No or not sure	1 [reference]	1 [reference]	1 [reference]	1 [reference]	1 [reference]	1 [reference]
Yes	1.64 [1.20–2.24]**	1.10 [0.79–1.53]	1.30 [1.04–1.64]*	0.84 [0.64–1.10]	1.36 [1.09–1.68]**	1.52 [1.27–1.82]***
Number of children ever born						
0	1 [reference]	1 [reference]	1 [reference]	1 [reference]	1 [reference]	1 [reference]
1–3	1.04 [0.83–1.32]	0.89 [0.63–1.27]	0.98 [0.78–1.21]	0.97 [0.72–1.31]	1.15 [0.96–1.37]	1.09 [0.94–1.27]
4+	1.116 [0.84–1.60]	0.93 [0.60–1.42]	1.08 [0.82–1.42]	0.84 [0.60–1.17]	1.28 [0.10–1.61]	1.11 [0.91–1.34]
Currently breastfeeding						
No	1 [reference]	1 [reference]	1 [reference]	1 [reference]	1 [reference]	1 [reference]
Yes	1.14 [0.90–1.45]	1.11 [0.88–1.41]	1.09 [0.93–1.29]	0.43 [0.23–0.79]**	1.08 [0.95–1.23]	1.09 [0.98–1.21]
Current method of contraceptives						
No method	1 [reference]	1 [reference]	1 [reference]	1 [reference]	1 [reference]	1 [reference]
Folkloric method	2.97 [1.07–8.28] *	0.41 [0.09–1.84]	1.87 [0.86–4.08]	1.05 [0.53–2.10]	1.57 [0.81–3.03]	1.37 [0.84–2.24]
Traditional	1.18 (0.87–1.60)	0.96 [0.64–1.44]	1.12 [0.88–1.44]	1.71 [0.61–4.79]	0.48 [0.14–1.62]	0.96 [0.45–2.03]
Modern	0.85 (0.68–1.06)	0.78 [0.60–1.01]	0.79 [0.67–0.93]**	0.77 [0.57–1.04]	0.75 [0.64–0.90]**	0.77 [0.66–0.90]**
Reads newspaper						
No	1 [reference]				1 [reference]	1 [reference]
Yes	0.98 [0.83–1.19]				1.05 [0.90–1.40]	0.99 [0.83–1.18]
Watches television						
No	1 [reference]				1 [reference]	1 [reference]
Yes	0.93 [0.78–1.11]				0.89 (0.62–1.32)	0.90 [0.81–1.01]

*P < 0.05, **P < 0.01, ***P < 0.001. CI, Confidence Interval; cOR, Crude Odds Ratio; aOR, adjusted Odds Ratio.

#### Sierra Leone

In Sierra Leone period 1 analyses, women covered with health insurance had 57% [aOR: 0.43 95% CI: 0.23–0.79, *p* < 0.001] reduced odds of developing anaemia than those not covered with health insurance. In addition, in period 2 analyses, women aged 20–24 years had 29% [aOR: 0.71 95% CI:0.58–0.86, *p* < 0.01] reduced odds of developing anaemia against women aged 15–19 years in Sierra Leone. Women aged 25–29 years and 30–34 years also had 46% [aOR:0.64 95% CI:0.51–0.80, *p* < 0.05] and 43% [aOR:0.57 95% CI: 0.44–0.73, *p* < 0.01] reduced odds of anaemia, respectively, compared with women aged 15–19 years. Muslim women had 1.33% [95% CI:1.14–1.55, *p* < 0.001] increased odds of developing anaemia than Christian women. Pregnant women had 1.36% [95% CI: 1.09–1.68, *p* < 0.01] increased odds of experiencing anaemia than non-pregnant women in Sierra Leone. Women using modern methods of contraceptives also had 25% [aOR:0.75 95% CI:0.64–0.90, *p* < 0.01] reduced odds of developing anaemia compared with women not using any method of contraceptives.

Furthermore, women aged 20–24 years had 28% [aOR: 0.72 95% CI: 0.61–0.86, *p* < 0.001] reduced odds of developing anaemia than women aged 15–19 years in Sierra Leone. In addition, women aged 25–29 years and 30–34 years also had 29% [aOR: 0.71 95% CI: 0.59–0.85, *p* < 0.001] and 35% [aOR: 0.65 95% CI: 0.54–0.81, *p* < 0.001] reduced odds of developing anaemia compared with women aged 15–19 years. In addition, women aged 45–49 years also had 38% [aOR: 0.62 95% CI: 0.48–0.80, *p* < 0.001] reduced odds of experiencing anaemia compared with women aged 15–19 years. In addition, pregnant women had 1.52% [95% CI: 1.27–1.82, *p* < 0.001] increased odds of experiencing anaemia than non-pregnant women in Sierra Leone. Women using modern contraceptives also had 23% [aOR: 0.77 95% CI: 0.66–0.90, *p* < 0.01] reduced odds of developing anaemia compared with non-contraceptive users (Table 5).

#### Mali

With period 1 analyses, women aged 30–34 years and 35–39 years had 43% [aOR: 0.67 95% CI: 0.51–0.88, *p* < 0.01] and 43% [aOR: 0.67, 95% CI: 0.47–0.97, *p* < 0.05] reduced odds of developing anaemia, respectively, compared with women aged 15–19 years. Women within the richest wealth index had 32% [aOR: 0.68 95% CI: 0.50–0.92, *p* < 0.05] reduced odds of developing anaemia compared with women in the poorest wealth index. Women belonging to other religious groups had 46% [aOR: 0.54 95% CI: 0.36–0.82, *p* < 0.01] reduced odds of developing anaemia compared with those in the Christian groups. Additionally, pregnant women in Mali had 1.41% [95% CI: 1.11–1.79, *p* < 0.01] increased odds of developing anaemia compared with non-pregnant women. Women with 4+ parity had 1.43% [95% CI: 1.08–1.90, *p* < 0.05] increased odds of developing anaemia compared with women with zero parity.

In period 2, women with secondary level of education in Mali had 29% [aOR: 0.71 95% CI: 0.55–0.91, *p* < 0.01] reduced odds of experiencing anaemia compared to women with no education. Moreover, women within the richest wealth index had 49% [aOR:0.51 95% CI: 0.36–0.72, *p* < 0.001] reduced odds of experiencing anaemia compared with women in the poorest index and women using folkloric and traditional methods of contraceptives had 73% [aOR:0.27 95% CI:0.10–0.73, *p* < 0.05] and 81% [aOR:0.19 95% CI:0.04–0.99, *p* < 0.05] reduced odds of experiencing anaemia compared with women not using any contraceptive methods. In addition, women using modern contraceptives had 29% [aOR: 0.71 95% CI: 0.58–0.88, *p* < 0.01] reduced odds of experiencing anaemia compared with women not using any method.

With regard to the combined period analyses, women aged 35–39 years had 22% [aOR: 0.78 95% CI: 0.62–0.99, *p* < 0.05] reduced odds of developing anaemia compared with women aged 15–19 years. In addition, women within the richer and richest wealth category had 21% [aOR: 0.79 95% CI: 0.65–0.96, *p* < 0.05] and 45% [aOR: 0.55 95% CI: 0.44–0.70, *p* < 0.001] reduced odds of developing anaemia than women within the poorest wealth index. Furthermore, women reading newspapers had 21% [aOR: 0.79 95% CI: 0.64–0.97, *p* < 0.05] reduced odds of developing anaemia compared with women not reading newspapers. Pregnant women in Mali also had 1.31% [95% CI: 1.10–1.56, *p* < 0.01] increased odds of developing anaemia compared with non-pregnant women. Women using modern contraceptives had 23% [aOR: 0.77 95% CI: 0.66–0.90, *p* < 0.01] reduced odds of developing anaemia compared with non-contraceptive users (Table 6).

**Table 6:** Factors influencing anaemia among women of reproductive age, period 1, period 2, and all pooled data of Benin and Mali DHS.

	Mali			Benin		
Variables	Period 1	Period 2	Pooled data	Period 1	Period 2	Pooled data
	aOR (95% CI)	aOR (95%CI)	aOR (95%CI)	aORs (95% CI)	aORs (95%CI)	aORs (95%CI)
Age category						
15–19	1 [reference]	1 [reference]	1 [reference]	1 [reference]	1 [reference]	1 [reference]
20–24	0.85 [0.66–1.10]	0.87 [0.66–1.13]	0.86 [0.71–1.04]	0.92 [0.72–1.17]	0.91 [0.77–1.08]	0.86 [0.75–0.99]*
25–29	0.84 [0.64–1.10]	0.91 [0.70–1.19]	0.87 [0.72–1.07]	1.03 [0.77–1.36]	0.79 [0.63–0.98]*	0.78 [0.65–0.93]**
30–34	0.67 [0.51–0.88]**	0.89 [0.65–0.22]	0.77 [0.62–0.96]	0.92 [0.69–1.23]	0.83 [0.66–1.05]	0.75 [0.63–0.91]**
35–39	0.67 [0.47–0.97]*	0.92 [0.67–1.29]	0.78 [0.62–0.99]*	0.86 [0.62–1.18]	0.83 [1.08–0.65]	0.75 [0.61–0.92]**
40–44	0.65 [0.57–1.10]	0.81 [0.57–1.14]	0.81 [0.63–1.04]	1.06 [0.76–1.47]	1.05 [0.79–1.41]	0.91 [0.73–1.13]
45–49	0.79 [0.50–1.11]	0.82 [0.53–1.24]	0.78 [0.58–1.05]	1.03 [0.71–1.50]	0.83 [0.62–1.12]	0.84 [0.67–1.06]
Marital status						
Single			1 [reference]	1 [reference]	1 [reference]	1 [reference]
Married			0.99 [0.80–1.24]	1.16 (0.85–1.58)	0.94 [0.74–0.65]	1.07 [0.88–1.29]
Cohabiting			0.90 [0.61–1.35]	1.65 [1.19–2.29]**	1.19 [0.91–1.54]	1.34 [1.10–1.65]**
Separate/widowed/divorced			0.98 [0.69–1.39]	1.40 [0.95–2.07]	1.11 [0.82–1.49]	1.27 [1.01–1.61]*
Highest level of education						
No Education	1 [reference]	1 [reference]	1 [reference]	1 [reference]	1 [reference]	1 [reference]
Primary	0.88 [0.68–1.13]	0.83 [0.67–1.03]	0.91 [0.77–1.07]	1.04 [0.87–1.26]	1.02 [0.89–1.17]	1.09 [0.97–1.21]
Secondary	0.87 [0.65–1.16]	0.71 [0.55–0.91]**	0.83 [0.68–1.00]	0.86 [0.69–1.07]	0.90 [0.77–1.04]	1.99 [0.87–1.13]
Higher	0.85 [0.45–1.61]	0.76 [0.45–1.27]	0.92 [0.62–1.36]	1.05 [0.60–1.84]	1.03 [0.76–1.54]	1.29 [0.93–1.79]
Place of residence						
Urban	1 [reference]	1 [reference]	1 [reference]			
Rural	0.97 [0.78–1.22]	1.07 [0.81–1.42]	1.02 [0.84–1.22]			
Wealth index						
Poorest	1 [reference]	1 [reference]	1 [reference]	1 [reference]	1 [reference]	1 [reference]
Poorer	0.85 [0.67–1.00]	0.97 [0.76–1.24]	0.92 [0.79–1.07]	0.92 [0.76–1-12]	0.82 [0.69–0.97]*	0.86 [0.76–0.98]*
Mild	0.82 [0.68–1.07]	1.05 [0.81–1.37]	0.92 [0.78–1.10]	1.01 [0.83–1.23]	0.83 [0.69–1.00]	0.90 [0.79–1.04]
Richer	0.84 [0.65–1.08]	0.78 [0.58–1.04]	0.79 [0.65–0.96]*	0.84 [0.68–1.04]	0.76 [0.62–0.93]**	0.82 [0.70–0.95]**
Richest	0.68 [0.50–0.92]*	0.51 [0.36–0.72]***	0.55 [0.44–0.70]***	0.99 [0.78–1.26]	0.77 [0.62–0.95]*	0.89 [0.74–1.05]
Religion						
Christians	1 [reference]					1 [reference]
Moslems	0.76 [0.58–1.01]					1.21 [1.10–1.32]***
Traditionalists						
Others	0.54 [0.36–0.82]**					
Current pregnancy						
No or not sure	1 [reference]	1 [reference]	1 [reference]	1 [reference]	1 [reference]	1 [reference]
Yes	1.41 [1.11–1.79]**	1.21 [0.96–1.52]	1.31 [1.10–1.56]**	1.08 [0.87–1.34]	1.63 [1.37–1.96]***	1.43 [1.25–1.63]***
Number of children ever born						
0	1 [reference]	1 [reference]	1 [reference]	1 [reference]	1 [reference]	1 [reference]
1–3	1.08 [0.83–1.39]	0.90 [0.71–1.15]	0.95 [0.79–1.15]	0.75 [0.57–0.99]	1.17 [0.94–1.45]	0.96 [0.81–1.14]
4+	1.43 [1.08–1.90]*	0.91 [0.68–1.23]	1.14 [0.92–1.41]	0.72 [0.52–0.98]*	1.23 [0.92–1.63]	0.99 [0.80–1.23]
Currently breastfeeding						
No	1 [reference]	1 [reference]	1 [reference]	1 [reference]	1 [reference]	1 [reference]
Yes	0.98 [0.82–1.16]	0.92 [0.77–1.09]	1.08 [0.84–1.08]	1.13 [0.96–1.34]	0.98 [0.86–1.12]	1.07 [0.97–1.19]
Smoke cigarette						
No						1 [reference]
Yes						1.71 [1.03–2.83]*
Covered with health insurance						
No		1 [reference]	1 [reference]			1 [reference]
Yes		1.11 [0.83–1.49]	1.08 [0.87–1.36]			0.78 [0.55–1.10]
Current method of contraceptives						
No method	1 [reference]	1 [reference]	1 [reference]	1 [reference]	1 [reference]	1 [reference]
Folkloric method	0.86 [0.67–1.11]	0.27 [0.10–0.73]*	0.72 [0.35–1.46]	1.39 [0.84–2.30]	0.99 [0.73–1.33]	0.92 [0.56–1.52]
Traditional	0.83 [0.62–1.12]	0.19 [0.04–0.99]*	0.30 [0.07–1.22]	1.47 [1.06–2.05]*	0.77 [0.66–0.90]**	1.12 [0.89–1.40]
Modern	0.87 [0.46–1.64]	0.71 [0.58–0.88]**	0.77 [0.66–0.90]**	1.11 [0.87–1.41]		0.91 [0.81–1.03]
Reads newspaper						
No	1 [reference]	1 [reference]	1 [reference]			
Yes	0.76 [0.58–1.01]	0.94 [0.67–1.31]	0.79 [0.64–0.97]*			
Watches television						
No	1 [reference]	1 [reference]	1 [reference]			1 [reference]
Yes	0.97 [0.83–1.13]	0.95 [0.80–1.14]	1.08 [0.96–1.21]			0.88 [0.75–1.03]
Listens to radio						
No	1 [reference]	1 [reference]	1 [reference]			1 [reference]
Yes	0.92 [0.79–1.07]	0.93 [0.78–1.09]	0.90 [0.81–1.01]			0.94 [0.85–1.04]

*P < 0.05, **P < 0.01, ***P < 0.001. CI, Confidence Interval; cOR, Crude Odds Ratio; aOR, adjusted Odds Ratio.

#### Benin

With period 1, women cohabitating had 1.65% [95% CI: 1.19–2.29, *p* < 0.01] increased odds of experiencing anaemia compared with single women in Benin. In addition, women with 4+ parity had 28% [aOR: 0.72 95% CI: 0.52–0.98, *p* < 0.05] reduced odds of anaemia compared with women with zero parity. Women using traditional methods of contraceptives had 1.47% [95% CI: 1.06–2.05, *p* < 0.05] increased odds of experiencing anaemia compared with women not using any method in Benin. In Benin period 2, women aged 25–29 years had 29% [aOR: 0.71 95% CI: 0.58–0.88, *p* < 0.01] reduced odds of anaemia compared with women aged 15–19 years. Women in the poorer wealth index had 19% [aOR: 0.82 95% CI: 0.69–0.97, *p* < 0.05] reduced odds of developing anaemia than women within the poorest wealth index. Similarly, women in the richer and richest wealth index had 24% [aOR: 0.76 95% CI: 0.62–0.93, *p* < 0.01] and 23% [aOR: 0.77 95% CI: 0.62–0.95, *p* < 0.05] reduced odds of developing anaemia, respectively, compared with women in the poorest category. Additionally, pregnant women were 1.63% [95% CI: 1.37–1.96, *p* < 0.001] more odds of developing anaemia compared with non-pregnant women. In addition, women using traditional methods of contraceptives had 23% [aOR: 0.77 95% CI: 0.66–0.90, *p* < 0.01] reduced odds of developing anaemia compared with women not using any contraception methods. Furthermore, for all survey periods, in the combined analysis, women aged 20–24 years had 14% [aOR: 0.86 95% CI: 0.75–0.99, *p* < 0.05] reduced odds of developing anaemia compared with women aged 15–19 years. Moreover, within the age categories of participants, women aged 25–29 years and 30–34 years also had 22% [aOR: 0.78 95% CI: 0.65–0.93, *p* < 0.01] and 25% [aOR: 0.75 95% CI: 0.63–0.91, *p* < 0.01] reduced odds of developing anaemia compared with women aged 15–19 years. Moreover, women cohabitating with a man had 1.34% [95% CI: 1.10–1.65, *p* < 0.01] and, similarly, separated/widowed/divorced women also had 27% [aOR: 1.27 95% CI: 1.01–1.61, *p* < 0.05] increased odds of developing anaemia compared with single women. Women within the poorer and richer wealth categories had 14% [aOR: 0.86 95% CI: 0.76–0.98, *p* < 0.05] and 18% [aOR: 0.82 95% CI: 0.70–0.95, *p* < 0.01] reduced odds of developing anaemia, respectively, compared with women within the poorest wealth category. Additionally, pregnant women also had 43% [aOR: 1.43 95% CI: 1.25–1.63, *p* < 0.001] increased odds of developing anaemia compared with non-pregnant women (Table 6).

### Endowments and coefficients in decomposition analysis

The decomposition analysis in Ghana showed that the decrease was mostly due to behaviour change. The decrease explained by the coefficient effects or behavioural changes in the selected explanatory variables accounted for −0.17 [*p* < 0.001], representing 99.7% of the total change compared with the part explained by the changes in the endowment. Regarding the total percentage decrease in anaemia attributable to the changes in coefficients, the independent variables providing significant contributions were place of residence, wealth index, and methods of contraceptives. The change in the effect of women living in rural areas contributed to the reduction in anaemia by −0.06 [*p* < 0.001], representing 34.5%. Moreover, the change in the effect of women in the richest wealth index was 0.04 [*p* < 0.05], approximately 22.7% of reduction in anaemia. The change in the effect of women using folkloric contraceptives accounted to −0.003 [*p* < 0.05], representing almost 2% of the overall reduction in anaemia. The decrease (0.94%) explained by the change in endowments was insignificant (Supplementary Table E).

With regard to the analysis performed with Mali datasets, the increase in anaemia was explained by the coefficient effects or behavioural changes in the selected explanatory variables was 0.13% [*p* < 0.001], representing 110.8% of the total change while the part explained by the changes in the endowment was −0.01% [*p* < 0.01], representing −10.8%. The independent variables providing significant contributions were educational level, wealth index, religion, parity, and use of contraceptives (Supplementary Table F).

The increase explained by the coefficient effects or behavioural changes in the selected explanatory variables was 0.16% [*p* < 0.001] in Benin, representing 100.61% of the total change than the part explained by changes in the endowment. The intercept accounted for 0.23%, [*p* < 0.001] representing 139.41% of the overall increase. This probably suggests that the model fit presented some limitations in explaining the increase in anaemia between the two survey periods. The independent variables providing significant contributions were listening to radio, parity, and pregnancy status (Supplementary Table G).

Finally, Sierra Leone decomposed datasets showed that the part explained by changes in the endowment was −0.01% [*p* < 0.05], representing 185.1% of the total change. The independent variables providing significant contributions were current contraceptive methods, religion, wealth index, residence, marital status, and age of women. The decrease (−85.1) explained by the change in coefficient was insignificant and intercept (Supplementary Table H).

## Discussion

This study examined the trends of anaemia prevalence, its consistent predictors, and factors contributing to these changes among women of reproductive age in Ghana, Mali, Benin, and Sierra Leone. The study observed that Sierra Leone and Ghana experienced a decline in anaemia prevalence over the periods. The decrease in Ghana is drastic while the decrease in Sierra Leone is marginal. There was an overall significant decline of 17.07% in anaemia in Ghana and 1.0% decrease in Sierra Leone from period 1 to period 2. Benin and Mali experienced worsened anaemia prevalence over the periods. Mali had a large trend significant increase of 11% in anaemia prevalence (period 1 to period 2), while Benin had the largest trend significant increase of 16.7% in anaemia (period 1 and period 2). The drastic reduction in anaemia prevalence observed in Ghana results from concerted efforts by the government of Ghana with its global health development partners through large-scale social programs. This has been largely done through targeted programs with the involvement of communities and vulnerable groups. Prioritized interventions greatly influencing this reduction include an increase in social safety net programs such as national health insurance, Livelihood Empowerment Against Poverty (LEAP), school feeding programs, folate supplement distribution to pregnant women and adolescent girls, and provision of sulfadoxine/pyrimethamine (SP) for malaria treatment during pregnancy (30).

Our findings generally support the large scale reported 50% anaemia in non-pregnant women and pregnant women in Central and West Africa (3). The observed trend estimates also corroborate studies pointing out that countries within SSA have the highest regional prevalence of anaemia with a slower decline over time (5, 31). In addition, the trend estimates affirm the findings associating West and Central African countries with the severe least improvement in reducing anaemia over time for sub-groups such as non-pregnant and pregnant women (3). These differential increases and decreases observed in countries across women characteristics may result from the multifaceted causal nature of anaemia. This issue could be supported by the observations made on the drivers of anaemia burden in SSA to be largely driven by the mixed effects of population growth, aging population, infectious diseases, and iron deficiency (5). Other causes of anaemia reported in low- and middle-income countries include iron deficiency, inflammations, and infections (11). The high burden of anaemia observed in Mali from this study may be attributed to the stagnated ITN usage and IFA supplementation among women, especially pregnant women as well as low deworming against infections as reported in the USAID 2010 Mali national anaemia profile (32).

### Factors significantly increasing and decreasing anaemia

Generally, across pooled data and the combined data estimates in all the countries, the odds of developing anaemia were significantly reduced across age categories. This finding corroborates what has been reported in Ghana, revealing high anaemia prevalence during the early stages of the reproductive age (15–29 years) with a decline towards the late periods of the reproductive age (40–49 years) (33). The finding also affirms similar findings observed in Ethiopia (34). Possessing health insurance in Sierra Leone (period 1) significantly reduced the odds of anaemia. There is a grey literature highlighting the specific effect of health insurance on anaemia; however, health insurance provides sustainable access to health service, which may influence general health outcomes in the population. Period 1, Period 2, and pooled data estimate from Mali, and pooled data from Ghana revealed that women in the poorest wealth index have increased odds of anaemia compared with those in relatively higher wealth index categories. This finding is in-line with other studies observing similar inequity findings (15, 16, 18, 34). In addition, a study analysing anaemia burden in low- and middle-income countries showed anaemia to be disproportionately concentrated in low socioeconomic groups (12). The analyses of period 1 and period 2 datasets in Benin revealed that women cohabitating and those separated/divorced/widowed had higher chances of being anaemic compared with single women. This finding is affirmed by an analysis of the 2015–2016 Myanmar Demographic and Health Survey, which also associated being married with higher odds of experiencing anaemia (35). The findings from the analysis of Mali (period 2) and Ghana (pooled data) datasets showed that women with secondary level of education and those with higher level of education have reduced odds of developing anaemia. In support of this finding, a study conducted in Timor-Leste (19) also observed illiteracy to be significantly associated with increased odds of experiencing anaemia (19). The study finding is in-line with the similar studies conducted (18, 34, 36). Women reading newspapers in Mali (pooled data) had reduced risk of developing anaemia, and this may be because of segments of news bulletins and advertisements, often projecting good nutritional habits and practices. Furthermore, in Benin (pooled data), women who smoke had increased odds of developing anaemia. However, a study conducted in India contradicts this study finding (37). In addition, smoking habit has been identified to cause diseases associated with anaemia, though the resulting low haemoglobin levels may be counterbalanced with increased red blood cell production because of chronic exposure to carbon monoxide (38). Almost across all survey periods in all countries, pregnant women consistently had higher odds of experiencing anaemia compared with non-pregnant women. This study findings is in line with findings from Ethiopia, Mali and Myanmar (18, 34, 35). Period 2 analysis in Mali showed that women using traditional methods of contraceptives have reduced odds of experiencing anaemia compared with women not using any methods. However, Benin (period 1 and period 2) analysis showed that women using traditional methods of contraceptives had increased odds of being anaemic. In addition, in Ghana (period 1) and Mali (period 2), women using folkloric methods of contraceptives had increased odds of developing anaemia. This study has, therefore, revealed contrasting findings across periods in countries regarding different types of contraceptive use and its effects on anaemia. Moreover, women using modern contraceptives had reduced odds of experiencing anaemia compared with women not using contraceptives in all survey periods across all countries. This finding is similar to studies conducted in Nepal and SSA (39, 40). Furthermore, period 1 estimates of Mali datasets showed that women with 4+ parity had increased odds of experiencing anaemia, though in Benin (period 1), women with 4+ parity had reduced odds of being anaemic. However, high parity index has been identified elsewhere to be associated with a higher prevalence of anaemia (16). Relatedly, an association is observed between high gravidity and increased odds of anaemia (34).

### Endowment and coefficient in decomposition

This study further examined the differences in anaemia across the two survey periods in each country, to explain how compositional changes and behavioural effects of women characteristics affected the changes in anaemia prevalence observed in the countries. Behavioural effects explained the decrease in Ghana while endowments or compositional changes explained the decrease in Sierra Leone. Reduction in Ghana is significantly explained by behavioural effects, resulting from the immense advocacy and programs carried out in the country over the last decades. Historically, a comprehensive micronutrient and health (MICAH) program introduced by World Vision in 1995 was implemented in Ghana (41). The program was implemented from 1996 to 2005 with specific prudent measures, encompassing increased intake and bioavailability of micronutrients, such as iron, iodine, and vitamin A, reducing prevalence of diseases, such as diarrhoea, parasitic, and vaccine-preventable diseases, and enhancing the local capacity for delivery systems to improve micronutrient status. Other policy initiatives over the period in Ghana include the integrated Anaemia Control Strategy 2003, Strengthening Partnerships, Results, and Innovations in Nutrition Globally (SPRING) Project 2003, National Malaria Control Programme (NMCP) 2008–2015, and Ghana’s National Nutrition Policy (NNP) (Government of Ghana 2013). Specifically, malaria directly causes anaemia by destroying red blood cells and also indirectly decreases the production of new red blood cells (42). Significant variables observed in Ghana have behavioural effects, such as place of residence, wealth index, and methods of contraceptives. The decrease (0.94%) explained by the change in the composition in Ghana was insignificant. This probably resulted from insignificant structural composition changes in the population of women in the two survey periods. However, structural compositional variables explaining the decrease in Sierra Leone include current contraceptive methods, religion, wealth index, residence, marital status, and age of women. Benin and Mali experienced a drastic increase in anaemia prevalence across the two periods. The increased anaemia in these two countries was significantly explained by behavioural changes. The independent variables in Benin providing these significant behavioural effect contributions were listening to radio, parity, and pregnancy status, while in Mali, the independent variables providing significant behavioural effects were educational level, wealth index, religion, parity, and use of contraceptives. The intercept accounted for 0.23% [*p* < 0.001] overall increase in Benin. This probably suggests that the model fit presented some limitations in explaining the increase in anaemia between the two survey periods. The part explained by changes in structural composition in Mali was significant, suggesting significant composition changes in the population of women in the two periods.

## Conclusion

The findings of this study highlight differential changes in anaemia prevalence over the two survey periods and their combined data across sub-groups. The study found that Ghana and Sierra Leone had experienced a decrease in the anaemia trend from period 1 to period 2, while Mali and Benin experienced a significant increase in the anaemia trend from period 1 to period 2. Overall, the burden of anaemia in the countries is high and remains a serious nutritional challenge requiring imperative attention. Factors identified to be associated with anaemia in the study include age, possessing health insurance, belonging to a relatively higher wealth index category, marital status, parity, breastfeeding, contraceptive use, and pregnancy status. Furthermore, the study observed that behavioural effects of variables included in the study explained the significant decrease in anaemia in Ghana while in Sierra Leone, endowments or compositional changes in women characteristics explained the significant decrease. In addition, the observed anaemia increases in Mali and Benin were significantly explained by behavioural effects of women’s characteristics.

The study recommends that national surveys should encompass measures, such as prevalence infections (helminths, malaria etc.), non-specific inflammation, and micronutrient deficiencies to enhance a better understanding of the drivers of anaemia among countries in SSA, since, to the best of our knowledge, current national survey data on micronutrient deficiencies or helminth infections are unavailable. We also observed that anaemia reduction in Ghana and Mali was largely due to changes in women behaviours; therefore, programmes or advocacies geared towards individual uptake of good nutritional behaviours should be further encouraged and enhanced. In addition, there is the need for continuous monitoring of Hb levels of pregnant women during antenatal care (ANC) visit with adequate diet supplementation of iron-rich foods since the study observed that pregnant women have increased odds of being anaemic. Finally, we recommend further studies to properly quantify the association of modern contraceptives with anaemia since the use of modern contraceptives was significantly identified to reduce the odds of developing anaemia.

## Strengths and limitations

- We utilized nationally representative samples with high quality enabling the generalizability of study findings in sub-Saharan Africa (SSA).
- The study employed new methods (multivariate decomposition) to understand the factors that can be targeted in anaemia control efforts.
- Observations with complete datasets eligible for the study were large.
- The study data were obtained through a cross-sectional study design, hence preventing the inferences of causations.
- Factors identified to predict anaemia, such as micronutrient deficiencies, malaria, and helminth infections, were not included in the logistic regression and multivariable decomposition analyses due to lack of data.

## Data availability statement

Publicly available datasets were analyzed in this study. This data can be found at: http://dhsprogram.com/data/available-datasets.cfm.

## Ethics statement

The studies involving humans were approved by the Ghana Health Service Ethical Review Committee. The studies were conducted in accordance with the local legislation and institutional requirements. The participants provided their written informed consent to participate in this study.

## Author contributions

CG conceptualized the study. MS and CG led the data extraction, performed formal analysis, and interpreted and wrote the first draft of the manuscript. FD-C contributed to writing some of the sections of the manuscript. CG, MS, and FD-C reviewed the draft manuscript and contributed to the final version of the manuscript. All authors read and approved the final manuscript before submission.
