# Supplementary material for: Anaemia among women of reproductive age in selected sub-Saharan African countries: multivariate decomposition analyses of the demographic and health surveys data 2008–2018
Source: Front Public Health. 2024 Jan 5;11:1128214. doi: 10.3389/fpubh.2023.1128214 (PMC10799556; doi:10.3389/fpubh.2023.1128214)
Supplement: Supplementary file 1 [file Data_Sheet_1.docx]

**Supplementary Material**

**Anaemia among women of reproductive age in sub -Saharan Africa countries: Multivariate decomposition analyses of the demographic and health surveys data, 2008 – 2018**

Mohammed Gazali Salifu^1^; Frances Baaba da-Costa Vroom^2^ and Chris Guure^2*^

^1^Department of International Health, Bloomberg School of Public Health, The Johns Hopkins University, Baltimore, Maryland.

^2^Department of Biostatistics, School of Public Health, College of Health Sciences, University of Ghana - Legon, Accra

***Corresponding Author:** Chris Guure

Department of Biostatistics, School of Public Health**,** College of Health Sciences, University of Ghana**.** Email: [cbguure@ug.edu.gh](mailto:cbguure@ug.edu.gh)

Changes in the distribution of anaemia prevalence across women’s characteristics among the Four (4) sub-Saharan Africa countries.

In Ghana (*Supplementary* **Table A**), most of the changes were observed with women characteristics such as age of women, marital status, educational level, residence, wealth index, working status, and coverage with health insurance. Single women anaemia prevalence dropped marginally by 5.35% from period 1 to period 2. Anaemia prevalence decreased by 3.85% from period 1 to period 2 among women with primary level of education. Furthermore, anaemia prevalence increased largely by 7.81 percentage points among women in urban areas.

Changes in women characteristics regarding anaemia prevalence observed in Mali included women’s age, levels of education, wealth index, and women’s working status. Women with no education anaemia prevalence decreased by 8.04% from period 1 and period 2 likewise women in the poorest wealth category anaemia prevalence reducing by 3.82% from period 1 to period 2. (*Supplementary,* **Table B**). Also, women not using any method of contraceptives anaemia prevalence reduced by 5.58%.

Distributional changes in anaemia prevalence were also observed across women characteristics such as women’s age, marital status, highest education, residence, religion, and working status in Benin from period 1 to period 2. Also, the largest changes were observed with women cohabitating, women with secondary level of education, and women not currently working. Women working anaemia prevalence reduced largely by 15.97% from period 1 to period 2 likewise women with secondary level of education anaemia prevalence also declining by 27.6% from period 1 to period 2 (Supplementary, **Table C**).

Larger changes in anaemia prevalence distribution were observed in Sierra Leone. Also, single women anaemia prevalence increased by 7.13% while women cohabitating anaemia prevalence dropped by 7.61% from period 1 to period 2. In addition, women with no education, anaemia prevalence reduced by 7.61%. Furthermore, women who have 1-3 parity anaemia prevalence reduced by 7.51% from period 1 to period 2. Also, women not using any method of contraceptives anaemia prevalence reduced by 7.51% while those using modern contraceptives anaemia prevalence increased greatly by 9.18% from period 1 to period 2 (Supplementary, **Table D**).

Supplementary Table A**:** **Distribution and bivariate analysis of factors associated with anaemia prevalence Period 1, Period 2 & all Periods, Ghana DHS**.

|  | Period 1 | | | Period 2 | | | all Period | | | % change in anaemia |
| --- | --- | --- | --- | --- | --- | --- | --- | --- | --- | --- |
| Anaemia status | Anaemia (%) | No anaemia (%) | Pearson’s chi-square | Anaemia (%) | No anaemia (%) | Pearson’s chi-square | Anaemia (%) | No anaemia (%) | Pearson’s chi-square |  |
| Age category |  |  | [14.96]* |  |  | [34.75] ** |  |  | [48.80]*** |  |
| 15-19 | 555 (26.42) | 335 (21.48) |  | 398 (23.41) | 434 (18.6) |  | 953(25.16) | 769(19.79) |  | -3.01 |
| 20-24 | 403 (19.5) | 276 (19.46) |  | 323 (21.34) | 353 (16.67) |  | 726(20.27) | 629(17.82) |  | 1.84 |
| 25-29 | 327 (16) | 264 (19.15) |  | 209(15.36) | 379 (18.4) |  | 536(1.73) | 643(18.71) |  | -0.64 |
| 30-34 | 221 (11.39) | 180 (12.36) |  | 182 (14.03) | 311 (15.45) |  | 403(12.5) | 491(14.17) |  | 2.64 |
| 35-39 | 239 (12.04) | 173 (12.1) |  | 150(10.9) | 247(13.46) |  | 389(11.56) | 420(12.9) |  | -1.14 |
| 40-44 | 160 (8.083) | 117 (8.014) |  | 128(8.787) | 187(9.295) |  | 288(8.38) | 304(8.77) |  | -0.71 |
| 45-49 | 139 (6.559) | 99 (7.428) |  | 79 (6.17) | 157(8.129) |  | 218(6.40) | 256(7.84) |  | -0.39 |
| Marital status |  |  | [1.18] |  |  | [11.23] * |  |  | [6.62] |  |
| Single | 806 (39.19) | 557 (37.77) |  | 643 (40.62) | 781(36.33) |  | 1449 (39.79) | 1338 (36.92) |  | 1.43 |
| Married | 799 (38.23) | 554 (38.43) |  | 475 (32.88) | 819(38.16) |  | 1274(35.99) | 1373(38.27) |  | -5.35 |
| Cohabiting | 262 (13.69) | 207 (14.77) |  | 204 (15.44) | 275(15.13) |  | 466(14.42) | 482(14.98) |  | 1.75 |
| Separate/Widowed/Divorced | 177 (8.887) | 126 (9.028) |  | 147 (11.05) | 193(10.38) |  | 324(9.80) | 319(9.82) |  | 2.16 |

*Supplementary Table A*: Distribution and bivariate analysis of factors associated with anaemia prevalence Period 1, Period 2 & all Periods, Ghana DHS(Continuation).

|  | Period 1 | | | Period 2 | | | all Periods | | | % change in anaemia |
| --- | --- | --- | --- | --- | --- | --- | --- | --- | --- | --- |
| Anaemia status | Anaemia (%) | No anaemia (%) | Pearson’s chi-square | Anaemia (%) | No anaemia (%) | Pearson’s chi-square | Anaemia (%) | No anaemia (%) | Pearson’s chi-square |  |
| Highest level of education |  |  | [26.94]*** |  |  | [12.08]* |  |  | [46.21]*** |  |
| None |  |  |  |  |  |  |  |  |  |  |
| Primary | 602 (28.04) | 357 (22.74) |  | 387 (24.19) | 499 (21.44) |  | 989(26.43) | 856(21.98) |  | -3.85 |
| Secondary | 1375(68.57) | 994 (70.8) |  | 1006 (70.26) | 1408(70.25) |  | 2381(69.28) | 2402(70.47) |  | 1.69 |
| Higher | 67(3.387) | 93 (6.459) |  | 76 (5.546) | 161 (8.313) |  | 143(4.29) | 254(7.55) |  | 2.16 |
| Place of residence |  |  | [11.49]* |  |  | [0.01] |  |  | [9.32]* |  |
| Urban | 1001 (51.95) | 791 (57.75) |  | 794 (59.76) | 1172 (59.58) |  | 1795(55.22) | 1963(58.83) |  | 7.81 |
| Rural | 10043 (48.05) | 653 (42.25) |  | 675 (40.24) | 896 (40.42) |  | 1718(44.78) | 1549(41.17) |  | -7.81 |

*Supplementary Table A*: Distribution and bivariate analysis of factors associated with anaemia prevalence Period 1, Period 2 & all Periods, Ghana DHS(Continuation).

|  | Period 1 | | | Period 2 | | | All Periods | | | % change in anaemia |
| --- | --- | --- | --- | --- | --- | --- | --- | --- | --- | --- |
| Anaemia status | Anaemia (%) | No anaemia (%) | Pearson’s chi-square | Anaemia (%) | No anaemia (%) | Pearson’s chi-square | Anaemia (%) | No anaemia (%) | Pearson’s chi-square |  |
| Wealth Index |  |  | [12.77] |  |  | [29.69]** |  |  | [32.22]*** |  |
| Poorest | 258 (8.557) | 159 (7.433) |  | 238 (10.09) | 316 (9.645) |  | 496(9.2) | 475(8.73) |  | 1.53 |
| Poorer | 380 (18.25) | 234 (15.46) |  | 303 (18.18) | 322 (13.69) |  | 683(18.22) | 556(14.42) |  | -0.01 |
| Mild | 421 (21.36) | 289 (21.28) |  | 362 (24.43) | 446 (20.62) |  | 783(22.64) | 735(20.56) |  | 3.07 |
| Richer | 518 (26.52) | 359 (25.64) |  | 288 (22.41) | 500 (27.2) |  | 806(24.8) | 859(26.56) |  | -4.11 |
| Richest | 467 (25.31) | 403(30.18) |  | 278 (24.89) | 484 (28.84) |  | 745(25.14) | 887(29.4) |  | -0.42 |
| Religion |  |  | [6.57] |  |  | [2.09] |  |  | [9.62]* |  |
| Christians | 1676 (84.47) | 1220(86.21) |  | 1215 (86.05) | 1732(86.98) |  | 2891(85.13) | 2952(86.67) |  | 1.58 |
| Moslems | 267 (10.96) | 169 (10.17) |  | 206 (10.87) | 285(10.7) |  | 473(10.92) | 454(10.48) |  | -0.09 |
| Traditionalists | 39 (1.756) | 31 (2.025) |  | 19 (1.254) | 22 (1.004) |  | 58(1.55) | 53(1.42) |  | -0.51 |
| Others | 62 (2.812) | 24 (1.596) |  | 29 (1.83) | 29 (1.314) |  | 91(2.40) | 53(1.43) |  | -0.98 |
| Currently working |  |  | [3.04] |  |  | [17.47]*** |  |  | [15.35]** |  |
| No | 614(29.17) | 402 (26.47) |  | 529(33.31) | 599(26.81) |  | 1143(30.90) | 1001(26.67) |  | 4.14 |
| Yes | 1430 (70.83) | 1042(73.53) |  | 940(66.69) | 1469 (73.19) |  | 2370(69.1) | 2511(73.33) |  | -4.14 |

*Supplementary Table A*: Distribution and bivariate analysis of factors associated with anaemia prevalence Period 1, Period 2 & all Periods, Ghana DHS(Continuation).

|  | Period 1 | | | Period 2 | | | all Periods | | | % change in anaemia |
| --- | --- | --- | --- | --- | --- | --- | --- | --- | --- | --- |
| Anaemia status | Anaemia (%) | No anaemia (%) | Pearson’s chi-square | Anaemia (%) | No anaemia (%) | Pearson’s chi-square | Anaemia (%) | No anaemia (%) | Pearson’s chi-square |  |
| Current pregnancy |  |  | [9.43] ** |  |  | [0.69] |  |  | [7.43]* |  |
| No or not sure | 1884(91.96) | 1363(94.64) |  | 1347(92.69) | 1944(1944) |  | 3231(92.26) | 3307(93.91) |  | 0.73 |
| Yes | 160 (8.041) | 81 (5.362) |  | 122(7.314) | 124(6.596) |  | 282(7.74) | 205(6.09) |  | -0.73 |
| Children ever born |  |  | [1.22] |  |  | [6.82] |  |  | [5.82] |  |
| 0 | 819(39.69) | 584(39.39) |  | 607(38.96) | 742(34.76) |  | 1426(39.38) | 1326(36.67) |  | -0.73 |
| 1-3 | 773 (38.17) | 564(39.75) |  | 569(40.56) | 865(42.65) |  | 1342(39.17) | 1429(41.45) |  | 2.39 |
| 4+ | 452 (22.14) | 296(20.85) |  | 293 (20.48) | 461(22.6) |  | 745(21.45) | 757(21.88) |  | -1.66 |
| Breastfeeding |  |  | [1.01] |  |  | [1.11] |  |  | [1.29] |  |
| Yes | 392(18.58) | 267(17.25) |  | 248(20.52) | 398(19.09) |  | 676(19.39) | 665(18.33) |  | 1.98 |
| No | 1652(81.42) | 1177(82.75) |  | 1185(79.48) | 1670 (80.91) |  | 2837(80.61) | 2847(81.67) |  | -1.98 |
| Smoke Cigarette |  |  | [0.32] |  |  | [1.44] |  |  | [1.07] |  |
| Yes | 3(.1342) | 3 (.2137) |  | 1(.0246) | 1(.1539) |  | 4(0.09) | 4(0.18) |  | -0.1 |
| No | 2041(99.87) | 1441(99.79) |  | 1468(99.98) | 2067(99.85) |  | 3509(99.91) | 3508(99.82) |  | 0.1 |

*Supplementary Table A*: Distribution and bivariate analysis of factors associated with anaemia prevalence Period 1, Period 2 & all Periods, Ghana DHS(Continuation).

|  | Period 1 | | | Period 2 | | | all Periods | | | % change in anaemia |
| --- | --- | --- | --- | --- | --- | --- | --- | --- | --- | --- |
| Anaemia status | Anaemia (%) | No anaemia (%) | Pearson’s chi-square | Anaemia (%) | No anaemia (%) | Pearson’s chi-square | Anaemia (%) | No anaemia (%) | Pearson’s chi-square |  |
| Health Insurance |  |  | [2.04] |  |  | [0.18] |  |  | [17.39] |  |
| Yes | 882 (40.97) | 663 (43.4) |  | 962(62.1) | 1376(62.8) |  | 1844(49.82) | 2039 (54.79) |  | 21.13 |
| No | 1162(59.03) | 781 (56.6) |  | 507(37.9) | 692 (37.2) |  | 1669(50.18) | 1473(45.21) |  | -21.13 |
| Current method of contraceptives |  |  | [11.18]* |  |  | [10.72] |  |  | [24.24]*** |  |
| No method | 1637(79.39) | 1120(77.16) |  | 1173(78.77) | 1523(74.36) |  | 2810(79.13) | 2643(75.52) |  | -0.62 |
| Folkloric method | 17(.8974) | 4(.332) |  | 3 (.1326) | 5(.3202) |  | 20(0.58) | 9 (0.33) |  | -0.77 |
| Traditional | 127(6.57) | 84(6.089) |  | 64(4.844) | 87(5.154) |  | 191(5.85) | 171(5.54) |  | -1.73 |
| Modern | 263(13.14) | 236 (16.41) |  | 229(16.25) | 453(20.17) |  | 492(14.45) | 689(18.62) |  | 3.11 |
| Reads Newspaper |  |  | [5.54]* |  |  | [0.09] |  |  | [0.11] |  |
| Yes | 544(27.76) | 447 (31.44) |  | 304(22.26) | 433(21.83) |  | 848(25.46) | 880(25.8) |  | -5.5 |
| No | 1500(72.24) | 997 (68.56) |  | 1165(77.74) | 1635(78.17) |  | 2665(74.54) | 2632(74.2) |  | 5.5 |

*Supplementary Table A*: Distribution and bivariate analysis of factors associated with anaemia prevalence Period 1, Period 2 & all Periods, Ghana DHS(Continuation).

|  | Period 1 | | | Period 2 | | | all Periods | | | % change in anaemia |
| --- | --- | --- | --- | --- | --- | --- | --- | --- | --- | --- |
| Anaemia status | Anaemia (%) | No anaemia (%) | Pearson’s chi-square | Anaemia (%) | No anaemia (%) | Pearson’s chi-square | Anaemia (%) | No anaemia (%) | Pearson’s chi-square |  |
| Watches Television |  |  | [5.95] * |  |  | [1.54] |  |  | [17.98]*** |  |
| Yes | 1372(70.35) | 1028(74.12) |  |  | 1124(80.75) | 1635 (82.38) | 2496(74.71) | 2663(78.97) |  | 10.4 |
| No | 672(29.65) | 416(25.88) |  |  | 345(19.25) | 433(17.62) | 1017(25.29) | 849(21.03) |  | -10.4 |
| Listens to Radio |  |  | [2.16] |  |  | [2.12] |  |  | [1.89] |  |
| Yes | 1800(88.83) | 1823(90.38) |  |  | 1245(85.44) | 1787(87.14) | 3045(87.41) | 3070(88.48) |  | -3.39 |
| No | 244(11.17) | 161(9.616) |  |  | 224(14.56) | 281(12.86) | 468(12.59) | 442 (11.52) |  | 3.39 |

Note: P<0.05=*, P<0.01=**, P<0.001=***

Supplementary Table B: **Distribution and bivariate analysis of factors associated with anaemia, Period 1 and Period 2, & all Period Mali DHS**

|  | Period 1 | | | Period 2 | | | all Periods | | | % change in anaemia |
| --- | --- | --- | --- | --- | --- | --- | --- | --- | --- | --- |
| Anaemia status | Anaemia (%) | No anaemia (%) | Pearson’s chi-square | Anaemia (%) | No anaemia (%) | Pearson’s chi-square | Anaemia (%) | No anaemia (%) | Pearson’s chi-square |  |
| Age category |  |  | [ 7.13] |  |  | [ 6.67] |  |  | [6.32] |  |
| 15-19 | 461(17.48) | 453(17.9) |  | 664 (19.79) | 349 (18.13) |  | 1125(18.75) | 802(18.03) |  | 2.31 |
| 20-24 | 476 (17.39) | 467(18.3) |  | 546 (16.9) | 377 (19.29) |  | 1022(17.12) | 844(18.74) |  | -0.49 |
| 25-29 | 549(20.69) | 482(19.1) |  | 585 (18.98) | 366 (19.48) |  | 1134(19.75) | 848(19.3) |  | -1.71 |
| 30-34 | 423(15.43) | 406(16.4) |  | 518 (16.04) | 294 (15.76) |  | 941(15.77) | 700(16.18) |  | 0.61 |
| 35-39 | 316 (11.39) | 300(12.3) |  | 408 (13.32) | 228 (12.73) |  | 724(12.45) | 528(12.51) |  | 1.93 |
| 40-44 | 264 (10.19) | 218(8.88) |  | 273 (8.491) | 153 (8.682) |  | 537(9.26) | 371(8.8) |  | -1.7 |
| 45-49 | 184 (7.428) | 161(6.82) |  | 205 (6.479) | 124 (5.928) |  | 389(6.91) | 285(6.44) |  | -0.95 |

*Supplementary Table B*: Distribution and bivariate analysis of factors associated with anaemia, Period 1 and Period 2, & all Period Mali DHS (Continuation)

|  | Period 1 | | | Period 2 | | | all Periods | | | % change in anaemia |
| --- | --- | --- | --- | --- | --- | --- | --- | --- | --- | --- |
| Anaemia status | Anaemia (%) | No anaemia (%) | Pearson’s chi-square | Anaemia (%) | No anaemia (%) | Pearson’s chi-square | Anaemia (%) | No anaemia (%) | Pearson’s chi-square |  |
| Marital status |  |  | [ 5.87] |  |  | [ 8.10] |  |  | [11.22]* |  |
| Single | 349 (12.72) | 363(14.3) |  | 503(14.38) | 335(16.81) |  | 852(13.63) | 698(15.4) |  | 1.66 |
| Married | 2223(83.99) | 2020(81.) |  | 2563(82.38) | 1463(79.38) |  | 4786(83.11) | 3483(80.66) |  | -1.61 |
| Cohabiting | 45 (1.523) | 51 (2.076) |  | 22 (0.64) | 12 (.5258) |  | 67(1.04) | 63(1.42) |  | -0.88 |
| Separate/Widowed/Divorced | 56 (1.765) | 53 (1.96) |  | 111(2.596) | 81 (3.286) |  | 167(2.22) | 134(2.52) |  | 0.8 |
| Highest level of education |  |  | [48.61] *** |  |  | [90.69]*** |  |  | [110.32]*** |  |
| No Education | 2087 (79.1) | 1738(71.9) |  | 2290(71.06) | 1168(59.51) |  | 4377(74.71) | 2906(66.24) |  | -8.04 |
| Primary | 243 (8.845) | 272(10.49) |  | 402(12.78 | 245 (13.8) |  | 645(11) | 517(11.89) |  | 3.93 |
| Secondary | 319 (11.21) | 432(16.75) |  | 465(14.76) | 430 (23.96) |  | 784(13.15) | 862(19.81) |  | 3.55 |
| Higher | 24 (.838) | 45 (1.576) |  | 42(1.395) | 48 (2.724) |  | 66(1.14) | 93(2.06) |  | 0.56 |

*Supplementary Table B*: Distribution and bivariate analysis of factors associated with anaemia, Period 1 and Period 2, & all Period Mali DHS (Continuation)

|  | Period 1 | | | Period 2 | | | all Periods | | | % change in anaemia |
| --- | --- | --- | --- | --- | --- | --- | --- | --- | --- | --- |
| Anaemia status | Anaemia (%) | No anaemia (%) | Chi-square test | Anaemia (%) | No anaemia (%) | Pearson’s chi-square | Anaemia (%) | No anaemia (%) | Pearson’s chi-square |  |
| Place of residence |  |  | [33.36]*** |  |  | [102.66] *** |  |  | [118.06]*** |  |
| Urban | 688(20.48) | 858(27.33) |  | 876(20.86) | 795 (33.72) |  | 1564(20.69) | 1653(30.04) |  | 0.38 |
| Rural | 1985(79.5) | 1629(72.6) |  | 2323(79.14) | 1096 (66.28) |  | 4308(79.31) | 2725(69.96) |  | -0.38 |
| Wealth Index |  |  | [65.25] *** |  |  | [162.39]*** |  |  | [202.63]*** |  |
| Poorest | 578(22.86) | 395(16.98) |  | 612 (19.04) | 292(13.81) |  | 1190(20.77) | 687(15.64) |  | -3.82 |
| Poorer | 505(18.84) | 400(17.46) |  | 674(22.41) | 272(17.04) |  | 1179(20.79) | 672(17.28) |  | 3.57 |
| Mild | 507(20.15) | 438(18.68) |  | 660(21.1) | 292(15.77) |  | 1167(20.67) | 730(17.44) |  | 0.95 |
| Richer | 486(19.65) | 461(20) |  | 695(20.44) | 454(22.12) |  | 1181(20.08) | 915(20.9) |  | 0.79 |
| Richest | 597(18.5) | 293(26.88) |  | 558(17.01) | 581(31.25) |  | 1155(17.68) | 1374(28.73) |  | -1.49 |
| Religion |  |  | [7.42]* |  |  | [ 8.79] |  |  | [1.84] |  |
| Christians | 123(5.264) | 90 (3.821) |  | 65(2.454) | 45 (3.01) |  | 188(3.73) | 135(3.48) |  | -2.81 |
| Moslems | 2475(91.73) | 2321(92.7) |  | 3039(93.43) | 1810(94.36) |  | 5514(92.66) | 4131(93.33) |  | 1.7 |
| Traditionalists |  |  |  |  |  |  |  |  |  |  |
| Others | 75(3.003) | 76 (3.608) |  | 95(4.12) | 36 (2.632) |  | 170(3.61) | 112(3.19) |  | 1.12 |

*Supplementary Table B*: Distribution and bivariate analysis of factors associated with anaemia, Period 1 and Period 2, & all Period Mali DHS (Continuation)

|  | Period 1 | | | Period 2 | | | all Periods | | | % change in anaemia |
| --- | --- | --- | --- | --- | --- | --- | --- | --- | --- | --- |
| Anaemia status | Anaemia (%) | No anaemia (%) | Pearson’s chi-square | Anaemia (%) | No anaemia (%) | Pearson’s chi-square | Anaemia (%) | No anaemia (%) | Pearson’s chi-square |  |
| Currently working |  |  | [0.06] |  |  | [3.30] |  |  | [0.01] |  |
| No | 1478 (55.68) | 1377(55.34) |  | 1691(45.85) | 976(43.23) |  | 3169(50.3) | 2353(50.2) |  | -9.83 |
| Yes | 1195(44.32) | 1110(44.66) |  | 1508(54.15) | 915(56.77) |  | 2703(49.7) | 2025(49.8) |  | 9.83 |
| Current pregnancy |  |  | [18.99]*** |  |  | [9.91]** |  |  | [27.45]*** |  |
| No or not sure | 2313(86.31) | 2252(90.23) |  | 2810(87.43) | 1718(90.36) |  | 5123(86.92) | 3970(90.28) |  | 1.12 |
| Yes | 360 (13.69) | 235(9.775) |  | 389(12.57) | 173(9.645) |  | 749(13.08) | 408(9.72) |  | -1.12 |
| Number of children ever born |  |  | [16.63] ** |  |  | [ 6.92] |  |  | [20.62]*** |  |
| 0 | 450(16.15) | 477(18.6) |  | 689(19.66) | 404 (19.45) |  | 1139(18.07) | 881(18.96) |  | 3.51 |
| 1-3 | 1016(37.44) | 1021(40.51) |  | 1158(35.16) | 765(38.67) |  | 2174(36.19) | 1786(39.73) |  | -2.28 |
| 4+ | 1207(46.42) | 989(40.89) |  | 1352(45.17) | 722(41.88) |  | 2559(45.74) | 1711(41.31) |  | -1.25 |
| Currently Breastfeeding |  |  | [0.06] |  |  | [0.89] |  |  | [0.20] |  |
| Yes | 961(36.42) | 904(36.08) |  | 1085(35.97) | 649(37.3) |  | 2046(36.17) | 1553(36.6) |  | -0.45 |
| No | 1712(63.58) | 1583(63.92) |  | 2114(64.03) | 1242(62.7) |  | 3826(63.83) | 2825(63.4) |  | 0.45 |

*Supplementary Table B*: Distribution and bivariate analysis of factors associated with anaemia, Period 1 and Period 2, & all Period Mali DHS (Continuation)

|  | Period 1 | | | Period 2 | | | All Periods | | | % change in anaemia |
| --- | --- | --- | --- | --- | --- | --- | --- | --- | --- | --- |
| Anaemia status | Anaemia (%) | No anaemia (%) | Pearson’s chi-square | Anaemia (%) | No anaemia (%) | Pearson’s chi-square | Anaemia (%) | No anaemia (%) | Pearson’s chi-square |  |
| Smoke Cigarette |  |  | [ 0.51] |  |  | [ 0.10] |  |  |  |  |
| Yes | 3(.1208) | 3(.061) |  | 28(.7041) | 14(.6281) |  | 31(0.41) | 17(0.34) | [0.39] | 0.58 |
| No | 2484(99.88) | 2670(99.94) |  | 3171(99.3) | 1877(99.37) |  | 5841(99.59) | 4361(99.66) |  | -0.58 |
| Covered with Health Insurance |  |  | [1.10] |  |  | [17.45]** |  |  | [10.35]* |  |
| Yes | 70 (2.929) | 82(3.443) |  | 124(4.185) | 119(6.876) |  | 194(3.62) | 201(4.9) |  | 1.26 |
| No | 2603(97.07) | 2405(96.56) |  | 3075(95.82) | 1772(93.12) |  | 5678(96.38) | 4177(95.10) |  | -1.26 |
| Current method of contraceptives |  |  | [34.01]*** |  |  | [62.98]*** |  |  | [64.67]*** |  |
| No method | 2453(91.92) | 2151(87.38) |  | 2808(86.34) | 1534(78.34) |  | 5261(88.87) | 3685(83.54) |  | -5.58 |
| Folkloric method | 13(.4676) | 9 (0.2539) |  | 7(.2787) | 13(0.85) |  | 20(0.36) | 22(0.50) |  | -0.19 |
| Traditional | 2(.0533) | 2(0.0862) |  | 4(0.12) | 10(0.65) |  | 6(0.09) | 12(0.33) |  | 0.07 |
| Modern | 205(7.554) | 325(12.28) |  | 380(13.26) | 334(20.17) |  | 585(10.68) | 659(15.63) |  | 5.71 |

*Supplementary Table B*: Distribution and bivariate analysis of factors associated with anaemia, Period 1 and Period 2, & all Period Mali DHS (Continuation)

|  | Period 1 | | | Period 2 | | | All Periods | | | % change in anaemia |
| --- | --- | --- | --- | --- | --- | --- | --- | --- | --- | --- |
| Anaemia status | Anaemia (%) | No anaemia (%) | Pearson’s chi-square | Anaemia (%) | No anaemia (%) | Pearson’s chi-square | Anaemia (%) | No anaemia (%) | Pearson’s chi-square |  |
| Reads Newspaper |  |  | [40.10]*** |  |  | [ 29.73]*** |  |  |  |  |
| Yes | 208(6.978) | 331(12.15) |  | 181(5.853) | 174(9.998) |  | 389(6.36) | 505(11.24) | [77.02]*** | -1.13 |
| No | 2465(93.02) | 2156(87.85) |  | 3018(94.15) | 1717(90) |  | 5483(93.64) | 3873(88.76) |  | 1.13 |
| Watches Television |  |  | [28.47]*** |  |  | [43.86]*** |  |  | [41.71]*** |  |
| Yes | 1305(46.12) | 1409(53.55) |  | 1807(60.88) | 1252(70.11) |  | 3112(54.19) | 2661(60.58) |  | 14.76 |
| No | 1368(53.88) | 1078(46.45) |  | 1392(39.12) | 639(29.89) |  | 2760(45.81) | 1717(39.42) |  | -14.76 |
| Listens to Radio |  |  | [15.63] ** |  |  | [15.71]*** |  |  | [27.84]*** |  |
| Yes | 1783(66.21) | 1783(66.21) |  | 2147(68.85) | 1357(74.1) |  | 3930(67.65) | 3149(72.5) |  | 2.64 |
| No | 890(33.79) | 890(33.79) |  | 1052(31.15) | 534(25.9) |  | 1942(32.35) | 1229(27.5) |  | -2.64 |

Note: P<0.05=*, P<0.01=**, P<0.001=***

Supplementary Table C**: Distribution and bivariate analysis of factors associated with anaemia prevalence Period 1, Period 2 & all Period Benin DHS**.

|  | Period 1 | | | Period 2 | | | All Period | | | % change in anaemia |
| --- | --- | --- | --- | --- | --- | --- | --- | --- | --- | --- |
| Anaemia status | Anaemia (%) | No anaemia (%) | Pearson’s chi-square | Anaemia (%) | No anaemia (%) | Pearson’s chi-square | Anaemia (%) | No anaemia (%) | Pearson’s chi-square |  |
| Age category |  |  | [ 5.03] |  |  | [ 10.55] |  |  | [7.17] |  |
| 15-19 | 351 (17.09) | 582 (17.11) |  | 952 (20.61) | 693 (20.89) |  | 1303(19.52) | 1221(19.13) |  | 3.52 |
| 20-24 | 331 (16.33) | 480 (16.6) |  | 841 (18.25) | 622 (18.39) |  | 1172(17.66) | 1102(17.56) |  | 1.92 |
| 25-29 | 415 (20.53) | 543 (18.62) |  | 813 (17.59) | 642 (19) |  | 1228(18.5) | 1185(18.82) |  | -2.94 |
| 30-34 | 330 (16.47) | 513 (17.17) |  | 651 (14.26) | 485 (14.32) |  | 981(14.95) | 998(15.65) |  | -2.21 |
| 35-39 | 262 (12.07) | 401 (13.52) |  | 548 (11.58) | 394 (11.59) |  | 810(11.73) | 795(12.49) |  | -0.49 |
| 40-44 | 232 (10.82) | 314 (10.3) |  | 437 (9.502) | 266 (7.58) |  | 669(9.91) | 580(8.84) |  | -1.32 |
| 45-49 | 145 (6.685) | 204 (6.69) |  | 376 (8.202) | 291 (8.235) |  | 521(7.73) | 495(7.52) |  | 1.51 |
| Marital status |  |  | [ 23.84] *** |  |  | [16.42]** |  |  | [28.15]*** |  |
| Single | 443 (22.19) | 690 (23.54) |  | 1044 (22.74) | 853 (25.25) |  | 1487(22.57) | 1543(24.45) |  | 0.55 |
| Married | 1077 (51.08) | 1674 (55.41) |  | 2570 (55.28) | 1918(56.08) |  | 3647(53.98) | 3592(55.77) |  | 4.2 |
| Cohabiting | 426(21.04) | 474 (15.9) |  | 732 (15.99) | 434 (13.25) |  | 1158(17.56) | 908(14.48) |  | -5.05 |
| Separate/Widowed/Divorced | 120 (5.693) | 145 (5.153) |  | 272 (5.991) | 188 (5.425) |  | 392(5.90) | 333(5.29) |  | 0.29 |

*Supplementary Table C*: Distribution and bivariate analysis of factors associated with anaemia prevalence Period 1, Period 2 & all Period Benin DHS(Continuation).

|  | Period 1 | | | Period 2 | | | All Period | | | % change in anaemia |
| --- | --- | --- | --- | --- | --- | --- | --- | --- | --- | --- |
| Anaemia status | Anaemia (%) | No anaemia (%) | Pearson’s chi-square | Anaemia (%) | No anaemia (%) | Pearson’s chi-square | Anaemia (%) | No anaemia (%) | Pearson’s chi-square |  |
| Highest level of education |  |  | [ 4.67] |  |  | [18.27] ** |  |  | [16.04]** |  |
| No Education | 1326 (61.43) | 1896(60.53) |  | 2633 (56.98) | 1804 (53.51) |  | 3959(58.36) | 3700(56.77) |  | -4.45 |
| Primary | 339 (17.2) | 450 (16.06) |  | 900 (19.72) | 661 (19.04) |  | 1239(18.94) | 1111(17.65) |  | 2.52 |
| Secondary | 368 (19.17) | 596(21.47) |  | 999(21.57) | 853 (25.52) |  | 1367(20.83) | 1449(23.64) |  | -27.6 |
| Higher | 33 (2.19) | 41 (1.937) |  | 86 (1.734) | 75 (1.931) |  | 119(1.87) | 116(1.93) |  | -0.46 |
| Place of residence |  |  | [ 0.02] |  |  | [ 0.09] |  |  |  |  |
| Urban | 852 (45.31) | 1231 (45.1) |  | 2019 (41.43) | 1466 (41.77) |  | 2871(42.63) | 2697(43.32) | [0.63] | -3.88 |
| Rural | 1214 (54.69) | 1752 (54.9) |  | 2599 (58.57) | 1927 (58.23) |  | 3813(57.37) | 3679(56.68) |  | 3.88 |
| Wealth Index |  |  | [ 5.46] |  |  | [25.31] ** |  |  | [22.88]** |  |
| Poorest | 402 (17.36) | 564(16.31) |  | 862 (17.59) | 504 (14.02) |  | 1264(17.2) | 1068(15.09) |  | 0.23 |
| Poorer | 392 (17.66) | 564 (17.92) |  | 830 (18.25) | 589 (17.82) |  | 1222(18.07) | 1153(17.87) |  | 0.59 |
| Mild | 452 (20.17) | 622 (18.96) |  | 928 (20.98) | 682 (20.41) |  | 1380(20.73) | 1304(19.73) |  | 0.81 |
| Richer | 405 (19.18) | 648 (21.64) |  | 947 (21.46) | 752 (23.26) |  | 1352(20.75) | 1400(22.51) |  | 2.28 |
| Richest | 415 (25.62) | 585 (25.17) |  | 1051 (21.73) | 866 (23.26) |  | 1466(22.94) | 1451(24.81) |  | -3.89 |

*Supplementary Table C*: Distribution and bivariate analysis of factors associated with anaemia prevalence Period 1, Period 2 & all Period Benin DHS(Continuation).

|  | Period 1 | | | Period 2 | | | All Period | | | % change in anaemia |
| --- | --- | --- | --- | --- | --- | --- | --- | --- | --- | --- |
| Anaemia status | Anaemia (%) | No anaemia (%) | Pearson’s chi-square | Anaemia (%) | No anaemia (%) | Pearson’s chi-square | Anaemia (%) | No anaemia (%) | Pearson’s chi-square |  |
| Religion |  |  | [2.80] |  |  | [3.94] |  |  | [2.36] |  |
| Christians | 1173 (58.55) | 1631 (56.97) |  | 2529 (54.71) | 1897(55.44) |  | 3702(55.9) | 3528(56.15) |  | -3.84 |
| Moslems | 436 (20.54) | 704 (22.46) |  | 1341(29.22) | 984 (29.86) |  | 177726.53) | 1688(26.42) |  | 8.68 |
| Traditionalists | 295(13.42) | 421 (13.41) |  | 428 (9.283) | 310 (8.941) |  | 723(10.57) | 731(11.02) |  | -4.19 |
| Others | 162 (7.494) | 227 (7.17) |  | 320 (6.785) | 202(5.76) |  | 482(7.01) | 429(6.42) |  | -0.7 |
| Currently working |  |  | [ 4.99] |  |  | [0.05] |  |  | [22.30]*** |  |
| No | 804 (40.32) | 1264 (43.47) |  | 1140(24.35) | 818 (24.15) |  | 1944(29.31) | 2082(33.14) |  | -15.97 |
| Yes | 1262(59.68) | 1719 (56.53) |  | 3478(75.65) | 2575(75.85) |  | 4740(70.69) | 4294(66.86) |  | 15.97 |
| Current pregnancy |  |  | [ 0.09] |  |  | [45.32]*** |  |  | [34.59]*** |  |
| No or not sure | 1858(90.25) | 2712(90.51) |  | 4020 (87.12) | 3113 (91.86) |  | 5878(88.09) | 5825(91.23) |  | -3.13 |
| Yes | 208 (9.752) | 271(9.491) |  | 598 (12.88) | 280 (8.138) |  | 806(11.91) | 551(8.77) |  | 3.13 |

*Supplementary Table C*: Distribution and bivariate analysis of factors associated with anaemia prevalence Period 1, Period 2 & all Period Benin DHS(Continuation).

|  | Period 1 | | | Period 2 | | | All Period | | | % change in anaemia |
| --- | --- | --- | --- | --- | --- | --- | --- | --- | --- | --- |
| Anaemia status | Anaemia (%) | No anaemia (%) | Pearson’s chi-square | Anaemia (%) | No anaemia (%) | Pearson’s chi-square | Anaemia (%) | No anaemia (%) | Pearson’s chi-square |  |
| Number of children ever born |  |  | [ 0.99] |  |  | [ 5.22] |  |  | [1.73] |  |
| 0 | 477 (23.83) | 699 (23.64) |  | 1143(24.57) | 892(26.56) |  | 1620(24.34) | 1591(25.21) |  | 0.74 |
| 1-3 | 802 (40.42) | 1124(39.29) |  | 1683(36.87) | 1258(36.92) |  | 2485(37.97) | 2382(38.02) |  | -3.55 |
| 4+ | 787 (35.75) | 1160(37.06) |  | 1792(38.56) | 1243(36.52) |  | 2579(37.69) | 2403(36.77) |  | 2.81 |
| Currently Breastfeeding |  |  | [ 1.45] |  |  | [1.07] |  |  | [0.16] |  |
| Yes | 558 (27.5) | 791 (25.98) |  | 1335 (28.63) | 1005 (29.69) |  | 1893(28.28) | 1796(27.97) |  | 1.13 |
| No | 1508 (72.5) | 2192 (74.02) |  | 3283(71.37) | 2388(70.31) |  | 4791(71.72) | 4580(72.03) |  | -1.13 |
| Smoke Cigarette |  |  | [ 0.01] |  |  | [ 2.57] |  |  |  |  |
| Yes | 3 (0 .1207) | 3 (0.11) |  | 69 (1.619) | 38(1.187) |  | 72(1.15) | 41(0.69 | [7.78]* | 1.5 |
| No | 2063 (99.88) | 2980 (99.89) |  | 4549(98.38) | 3355(98.81) |  | 6612(98.85) | 6335(99.31) |  | -1.5 |
| Covered with Health Insurance |  |  | [ 0.16] |  |  | [3.72] |  |  | [4.84]* |  |
| Yes | 32 (1.741) | 50 (1.894) |  | 40 (0.87) | 44 (1.315) |  | 72(1.14) | 94(1.58) |  | -0.87 |
| No | 2034(98.26) | 2933(98.11) |  | 4578(99.13) | 3349(98.69) |  | 6612(98.86) | 6282(98.42) |  | 0.87 |

*Supplementary Table C*: Distribution and bivariate analysis of factors associated with anaemia prevalence Period 1, Period 2 & all Period Benin DHS(Continuation).

|  | Period 1 | | | Period 2 | | | All Period | | | % change in anaemia |
| --- | --- | --- | --- | --- | --- | --- | --- | --- | --- | --- |
| Anaemia status | Anaemia (%) | No anaemia (%) | Pearson’s chi-square | Anaemia (%) | No anaemia (%) | Pearson’s chi-square | Anaemia (%) | No anaemia (%) | Pearson’s chi-square |  |
| Current method of contraceptives |  |  | [ 23.15] |  |  | [23.15]*** |  |  | [8.79] |  |
| No method | 1743(84.36) | 2591(86.56) |  | 3981(86.44) | 2827(82.76) |  | 5724(85.79) | 5418(84.53) |  | 2.08 |
| Folkloric method | 44 (1.769) | 45 (1.352) |  | ## | ## |  | 44(0.55) | 45(0.63) |  |  |
| Traditional | 103 (4.94) | 93 (3.38) |  | 134(2.92) | 96 (3.09) |  | 237(3.55) | 189(3.23) |  | -2.02 |
| Modern | 176 (8.94) | 254 (8.709) |  | 503(10.64) | 470(14.15) |  | 679(10.11) | 724(11.62) |  | 1.7 |
| Reads Newspaper |  |  | [ 1.13] |  |  | [ 4.67] |  |  | [15.53]** |  |
| Yes | 264(14.36) | 408 (15.44) |  | 397(8.344) | 332 (9.738) |  | 661(10.21) | 740(12.39) |  | -6.01 |
| No | 1802(85.64) | 2575 (84.56) |  | 4221 (91.66) | 3061(90.26) |  | 6023(89.79) | 5636(87.61) |  | 6.01 |
| Watches Television |  |  | [ 0.02] |  |  | [ 3.67] |  |  | [12.10]** |  |
| Yes | 941 (49.16) | 1348(49.38) |  | 1766 (37.67) | 1340(39.78) |  | 2707(41.23) | 2688(44.24) |  | -11.49 |
| No | 1125(50.84) | 1635(50.62) |  | 2852(62.33) | 2053(60.22) |  | 3977(58.77) | 3688(55.76) |  | 11.49 |
| Listens to Radio |  |  | [ 3.57] |  |  | [ 3.57] |  |  | [3.50] |  |
| Yes | 1299(63.16) | 1920(65.74) |  | 2648(57.53) | 1905(56.66) |  | 3947(59.27) | 3825(60.88) |  | -5.63 |
| No | 767 (36.84) | 1063(34.26) |  | 1970(42.47) | 1488 (43.34) |  | 2737(40.73) | 2551(39.12) |  | 5.63 |
|  |  |  |  |  |  |  |  |  |  |  |

Note: P<0.05=*, P<0.01=**, P<0.001=***

Supplementary Table D**: Distribution and bivariate analysis of factors associated with anaemia prevalence Period 1, Period 2 & all Period Sierra Leone DHS**

|  | Period 1 | | | Period 2 | | | All Period | | | % change in anaemia |
| --- | --- | --- | --- | --- | --- | --- | --- | --- | --- | --- |
| Anaemia status | Anaemia (%) | No anaemia (%) | Pearson’s chi-square | Anaemia (%) | No anaemia (%) | Pearson’s chi-square | Anaemia (%) | No anaemia (%) | Pearson’s chi-square |  |
| Age category |  |  | [12.78] |  |  | [25.44]** |  |  | [36.72]*** |  |
| 15-19 | 257(17.23) | 257(13.69) |  | 862(23.75) | 889(19.49) |  | 1119(21.86) | 1146(17.83) |  | 6.52 |
| 20-24 | 219(15.25) | 283(16.32) |  | 564(16.13) | 687(16.71) |  | 783(1.87) | 970(16.6) |  | 0.88 |
| 25-29 | 302(23.37) | 375(22.05) |  | 591(17.36) | 679(17.31) |  | 893(19.10) | 1054(18.66) |  | -6.01 |
| 30-34 | 187(13.21) | 250(13.94) |  | 472(14) | 606 (14.72) |  | 659(13.77) | 856(14.5) |  | 0.79 |
| 35-39 | 216(16.56) | 268(16.41) |  | 492(13.85) | 614(14.49) |  | 708(14.64) | 882(15.04) |  | -2.71 |
| 40-44 | 121(8.03) | 171(9.905) |  | 250(7.202) | 359(8.866) |  | 371(7.44) | 530(9.16) |  | -0.83 |
| 45-49 | 91(6.34) | 125(7.685) |  | 276(7.715) | 363(8.418) |  | 367(7.32) | 488(8.21) |  | 1.38 |
| Marital status |  |  | [7.75] |  |  | [ 14.09] |  |  | [3.54] |  |
| Single | 283(18.08) | 312(15.21) |  | 922(25.21) | 1217(28.37) |  | 1205(23.14) | 1529(24.61) |  | 7.13 |
| Married | 889 (66.41) | 1143(68.85) |  | 2256(65.24) | 2634(62.73) |  | 3145(65.58) | 3777(64.48) |  | -1.17 |
| Cohabiting | 139(10.03) | 153(9.054) |  | 86(2.421) | 109(2.882) |  | 225(4.63) | 262(4.65) |  | -7.61 |
| Separate/Widowed/Divorced | 82(5.478) | 121(6.884) |  | 243(7.128) | 237(6.018) |  | 325(6.65) | 358(6.27) |  | 1.65 |

Supplementary Table D: Distribution and bivariate analysis of factors associated with anaemia prevalence Period 1, Period 2 & all Period Sierra Leone DHS(Continuation)

|  | Period 1 | | | Period 2 | | | All Period | | | % change in anaemia |
| --- | --- | --- | --- | --- | --- | --- | --- | --- | --- | --- |
| Anaemia status | Anaemia (%) | No anaemia (%) | Pearson’s chi-square | Anaemia (%) | No anaemia (%) | Pearson’s chi-square | Anaemia (%) | No anaemia (%) | Pearson’s chi-square |  |
| Highest level of education |  |  | [ 3.51] |  |  | [57.81] *** |  |  | [51.26]*** |  |
| No Education | 897(68.65) | 1110(68.23) |  | 2028(59.03) | 2258(54.14) |  | 2925(61.82) | 3368(58.17) |  | -9.62 |
| Primary | 186(11.99) | 227(12.73) |  | 488(14.69) | 540(13.13) |  | 674(13.9) | 767(13.02) |  | 2.7 |
| Secondary | 287(17.99) | 346(16.88) |  | 924(24.53) | 1248(28.62) |  | 1211(22.64) | 1594(25.26) |  | 6.54 |
| Higher | 23(1.374) | 46(2.151) |  | 67(1.753) | 151(4.107) |  | 90(1.64) | 197(3.55) |  | 0.38 |
| Place of residence |  |  | [1.57] |  |  | [114.51]*** |  |  | [70.74]*** |  |
| Urban | 562(35.03) | 714(32.9) |  | 1214(29.47) | 1916(41.23) |  | 1803(31.08) | 2630(38.30) |  | -5.56 |
| Rural | 831(64.97) | 1015(67.1) |  | 2266(70.53) | 2281(58.77) |  | 3097(68.92) | 3296(61.15) |  | 5.56 |
| Wealth Index |  |  | [ 3.11] |  |  | [127.11]*** |  |  | [96.30]*** |  |
| Poorest | 248(18.11) | 314(18.64) |  | 706(20.4) | 686(16.1) |  | 954(19.73) | 1000(16.83) |  | 2.29 |
| Poorer | 257(21.13) | 294(18.95) |  | 660(20.73) | 658(17.65) |  | 917(20.84) | 952(18.02) |  | -0.4 |
| Mild | 262(20.02) | 313(19.9) |  | 677(20.7) | 700(17.94) |  | 939(20.5) | 1013(18.50) |  | 0.68 |
| Richer | 298(19.96) | 380(21.72) |  | 825(20.7) | 1017(19.46) |  | 1123(19.99) | 1397(20.11) |  | 0.74 |
| Richest | 328(20.78) | 428(20.79) |  | 639(18.17) | 1136(28.85) |  | 967(18.93) | 1564(26.54) |  | -2.61 |

Supplementary Table D: Distribution and bivariate analysis of factors associated with anaemia prevalence Period 1, Period 2 & all Period Sierra Leone DHS(Continuation)

|  | Period 1 | | | Period 2 | | | All Period | | | % change in anaemia |
| --- | --- | --- | --- | --- | --- | --- | --- | --- | --- | --- |
| Anaemia status | Anaemia (%) | No anaemia (%) | Pearson’s chi-square | Anaemia (%) | No anaemia (%) | Pearson’s chi-square | Anaemia (%) | No anaemia (%) | Pearson’s chi-square |  |
| Religion |  |  | [1.23] |  |  | [56.31] *** |  |  | [42.28]*** |  |
| Christians | 378(22.93) | 467(23.35) |  | 680(17.34) | 1029(24.35) |  | 1058(18.96) | 1496(24.06) |  | -5.59 |
| Moslems | 1008(76.4) | 1249(75.62) |  | 2815(82.36) | 3158 (75.44) |  | 3823(80.63) | 4407(75.49) |  | 5.96 |
| Traditionalists | 1(0.04) | 1(0.07) |  | 1(0.06) | 0(0) |  | 2(0.54) | 1(0.02) |  |  |
| Others | 7(0 .67) | 13(1.03) |  | 12(0 .30) | 10(0 .21) |  | 17(0.35) | 22(0.42) |  | -0.37 |
| Currently working |  |  | [0.15] |  |  | [4.58] |  |  | [2.77] |  |
| No | 403(26.54) | 491(25.93) |  | 1200(31.05) | 1476(33.35) |  | 1603(29.74) | 1967(31.23) |  | 4.51 |
| Yes | 990(73.46) | 1238(74.07) |  | 2307(68.95) | 2721(66.65) |  | 3297(70.26) | 3959(68.77) |  | -4.51 |
| Current pregnancy |  |  | [1.25] |  |  | [25.27] *** |  |  | [11.54]* |  |
| No or not sure | 1241(88.74) | 1533(87.43) |  | 3145(89.72) | 3902(92.93) |  | 4386(89.43) | 5435(91.36) |  | 0.98 |
| Yes | 152(11.26) | 196(12.57) |  | 362(10.28) | 295(7.071) |  | 514(10.57) | 491(8.64) |  | -0.98 |

Supplementary Table D: Distribution and bivariate analysis of factors associated with anaemia prevalence Period 1, Period 2 & all Period Sierra Leone DHS(Continuation)

|  | Period 1 | | | Period 2 | | | All Period | | | % change in anaemia |
| --- | --- | --- | --- | --- | --- | --- | --- | --- | --- | --- |
| Anaemia status | Anaemia (%) | No anaemia (%) | Pearson’s chi-square | Anaemia (%) | No anaemia (%) | Pearson’s chi-square | Anaemia (%) | No anaemia (%) | Pearson’s chi-square |  |
| Number of children ever born |  |  | [7.91] |  |  | [0.39] |  |  | [0.87] |  |
| 0 | 281(18.23) | 282(15.56) |  | 845(23.66) | 1048(24.17) |  | 1126(22.09) | 1330(21.71) |  | 5.43 |
| 1-3 | 616(45.16) | 783(43.34) |  | 1321(37.65) | 1580(37.73) |  | 1937(39.83) | 2363(39.33) |  | -7.51 |
| 4+ | 496(36.6) | 664(41.11) |  | 1341(38.69) | 1560(38.1) |  | 1837(38.09) | 2233(38.96) |  | 2.09 |
| Currently Breastfeeding |  |  | [ 0.05] |  |  | [16.51]** |  |  | [12.41]** |  |
| No | 1011(70.34) | 1264(70.72) |  | 2578(72.62) | 3248(76.66) |  | 3589(71.96) | 4512(74.96) |  | 2.28 |
| Yes | 382(29.66) | 465(29.28) |  | 929(27.38) | 949(23.34) |  | 1311(28.04) | 1414(25.04) |  | -2.28 |
| Smoke Cigarette |  |  | [5.86]* |  |  | [0.72] |  |  | [4.34] |  |
| No | 1316(95.1) | 1610(93.03) |  | 3372(95.55) | 4010(95.15) |  | 4688(95.42) | 5620(94.54) |  | 0.45 |
| Yes | 77(4.896) | 119(6.968) |  | 135(4.446) | 187(4.855) |  | 212(4.58) | 306(5.46) |  | -0.45 |
| Covered with Health Insurance |  |  | [8.70]** |  |  | [0.43] |  |  | [5.84]* |  |
| Yes | 25(1.03) | 53(2.44) |  | 36(0.86) | 50(1.005) |  | 4839(99.09) | 5823(98.58) |  | -0.17 |
| No | 1368(98.97) | 1676(97.56) |  | 3471(99.14) | 4147(98.99) |  | 61(0.91) | 103(1.416) |  | 0.17 |

Supplementary Table D: Distribution and bivariate analysis of factors associated with anaemia prevalence Period 1, Period 2 & all Period Sierra Leone DHS(Continuation)

|  | Period 1 | | | Period 2 | | | All Period | | | % change in anaemia |
| --- | --- | --- | --- | --- | --- | --- | --- | --- | --- | --- |
| Anaemia status | Anaemia (%) | No anaemia (%) | Pearson’s chi-square | Anaemia (%) | No anaemia (%) | Pearson’s chi-square | Anaemia (%) | No anaemia (%) | Pearson’s chi-square |  |
| Current method of contraceptives |  |  | [6.31] |  |  | [75.16]*** |  |  | [71.05]*** |  |
| No method | 1248(90.74) | 1517(88.91) |  | 2858 (82.58) | 3160 (75.07) |  | 4106(84.95) | 4677(79.02) |  | -8.16 |
| Folkloric method | 16(1.16) | 18(1.16) |  | 27(0.87) | 26 (0.56) |  | 43(0.96) | 44(0.73) |  | -0.29 |
| Traditional | 12(0.9018) | 11(0 .5293) |  | 6(0 .18) | 16(0 .57) |  | 18(0.40) | 27(0.56) |  | -0.72 |
| Modern | 117(7.197) | 183(9.41) |  | 616(16.36) | 995(23.8) |  | 733(13.71) | 1178(19.69) |  | 9.18 |
| Reads Newspaper |  |  | [ 0.84] |  |  | [17.69] ** |  |  | [16.39]** |  |
| Yes | 158(9.645) | 229(10.64) |  | 346(8.769) | 527(11.71) |  | 504(9.02) | 756(11.4) |  | -0.88 |
| No | 1235(90.35) | 1500(89.36) |  | 3161(91.23) | 3670(88.29) |  | 4396(90.98) | 5170(88.6) |  | 0.88 |
| Watches Television |  |  | [0.39] |  |  | [47.66]*** |  |  | [40.28]*** |  |
| Yes | 235 (14.37) | 306 (15.16) |  | 606(16.95) | 1003(23.33) |  | 841(16.2) | 1309(21) |  | 2.58 |
| No | 1158(85.63) | 1423(84.84) |  | 2901(83.05) | 3194(76.67) |  | 4059(83.80) | 4617(79) |  | -2.58 |
|  | **Listens to Radio** |  |  | [1.66] |  |  | [30.76] *** |  |  | [28.82]*** |
|  | **Yes** | 800 (54.89) | 1030(57.2) |  | 2135(59.39) | 2782(65.53) |  | 2935(58.09) | 3812(63.15) | 4.5 |
|  | **No** | 593 ( 45.11) | 699(42.8) |  | 1372(40.61) | 1415(34.47) |  | 1965(41.91) | 2114(36.85) | -4.5 |

**Endowments and Coefficients in Decomposition Analysis**

The decomposition analysis in Ghana showed that the decrease was mostly due to behaviour change. The decrease explained by the coefficient effects or behavioural changes of the selected explanatory variables was -0.17 [p<0.001], representing 99.7% of the total change than the part explained by changes in the endowment. Regarding the total percent decrease in anaemia attributable to the changes in coefficients, the independent variables providing significant contributions were place of residence, wealth index, and methods of contraceptives. The change in the effect of women living in rural areas contributed to the reduction in anaemia by -0.06[p<0.001], representing 34.5%. Also, the change in the effect of women in the richest wealth index contributed 0.04 [p<0.05] approximately 22.7% to the reduction in anaemia. The change in the effect of women using folkloric contraceptives contributed -0.003 [p<0.05], representing almost 2% to the overall reduction in anaemia. The decrease (0.94%) explained by the change in endowments was insignificant as well as the intercept (*Supplementary,* **Table** **E).**

With regards to the analysis performed with Mali datasets, the increase in anaemia was explained by the coefficient effects or behavioural changes of the selected explanatory variables was 0.13, [p<0.001] representing 110.8% of the total change whilst the part explained by changes in the endowment was -0.01 [p<0.01] representing -10.8%. The independent variables providing significant contributions were educational level, wealth index, religion, parity and use of contraceptives (*Supplementary*, **Table F).**

The increase explained by the coefficient effects or behavioural changes of the selected explanatory variables was 0.16 [p<0.001] in Benin, representing 100.61% of the total change than the part explained by changes in the endowment. The intercept accounted for 0.23, [p<0.001] representing 139.41% of the overall increase. This probably suggests that the model fit presented some limitations in explaining the increase in anaemia between the two survey periods. The independent variables providing significant contributions were listening to radio, parity, and pregnancy status (*Supplementary*, **Table G).**

Lastly, Sierra Leone decomposed datasets showed that the part explained by changes in the endowment was -0.01 [p<0.05], representing 185.1% of the total change. The independent variables providing significant contributions were current contraceptives method, religion, wealth index, residence, marital status, and age of women. The decrease (-85.1) explained by the change in coefficient was insignificant as well as the intercept (*Supplementary*, **Table H).**

| Anaemia occurrence | Difference due to characteristics(E) | | Difference due to coefficient(C) | |
| --- | --- | --- | --- | --- |
|  | Coefficient | Percent (%) | Coefficient | Percent (%) |
| Age category |  |  |  |  |
| 15-19 | 1 [reference] | 1[reference] | 1 [reference] | 1[reference] |
| 20-24 | -0.0006732 | 0.39932 | 0.014373 | -8.5258 |
| 25-29 | 0.000294 | -0.17439 | 0.0068513 | -4.064 |
| 30-34 | -0.0047378 | 2.8103 | 0.0040641 | -2.4107 |
| 35-39 | -0.00079379 | 0.47085 | -0.0020163 | 1.196 |
| 40-44 | -0.0013959 | 0.82799 | 0.001248 | -0.74026 |
| 45-49 | -0.0013996 | 0.83021 | -0.0023006 | 1.3646 |
|  |  |  |  |  |
| Marital status |  |  |  |  |
| Single | 1 [reference] | 1[reference] | 1 [reference] | 1[reference] |
| Married | -0.002606 | 1.5458 | 0.019081 | -11.318 |
| Cohabiting | 0.0010752 | -0.63776 | 0.012395 | -7.3525 |
| Separate/Widowed/Divorced | 0.0043712 | -2.5929 | 0.0078636 | -4.6644 |
|  |  |  |  |  |
| Highest level of education |  |  |  |  |
| None |  |  |  |  |
| Primary | 1 [reference] | 1[reference] | 1 [reference] | 1[reference] |
| Secondary | -0.00052983 | 0.31428 | 0.012873 | -7.636 |
| Higher | -0.0065814 | 3.9039 | 0.0027937 | -1.6571 |
|  |  |  |  |  |
| Place of residence |  |  |  |  |
| Urban | 1 [reference] | 1[reference] | 1 [reference] | 1[reference] |
| Rural | 0.013088 | -7.7636 | -0.05811*** | 34.469 |
| Wealth Index |  |  |  |  |
| Poorest | 1 [reference] | 1[reference] | 1 [reference] | 1[reference] |
| Poorer | -0.0025914 | 1.5371 | 0.0048719 | -2.8898 |
| Mild | 0.00036934 | -.21908 | 0.0003446 | -.20441 |
| Richer | 0.0026436 | -1.5681 | -0.038283* | 22.708 |
| Richest | 0.00036282 | -.21521 | -0.033583 | 19.92 |
| Religion |  |  |  |  |
| Christians | 1 [reference] | 1[reference] | 1 [reference] | 1[reference] |
| Moslems | -0.000080173 | 0.047556 | -0.0039087 | 2.3185 |
| Traditionalists | -0.00045151 | 0.26782 | 0.0016368 | -0.97091 |
| Others | -0.0010586 | 0.62793 | -0.0015989 | 0.94843 |
| Currently working |  |  |  |  |
| No | 1 [reference] | 1[reference] | 1 [reference] | 1[reference] |
| Yes | 0.0018059 | -1.0712 | -.022916 | 13.593 |
| Current pregnancy |  |  |  |  |
| No or not sure | 1 [reference] | 1[reference] | 1 [reference] | 1[reference] |
| Yes | -0.000024401 | 0.014474 | -0.0074993 | 4.4483 |
| Number of children ever born |  |  |  |  |
| 0 | 1 [reference] | 1[reference] | 1 [reference] | 1[reference] |
| 1-3 | -0.0021441 | 1.2718 | -0.017449 | 10.35 |
| 4+ | -0.000050811 | 0.030139 | -0.013176 | 7.8154 |
| Currently Breastfeeding |  |  |  |  |
| No | 1 [reference] | 1[reference] | 1 [reference] | 1[reference] |
| Yes | 0.0011508 | -.68264 | -0.0026788 | 1.589 |

Supplementary Table E**: Multivariable decomposition of characteristics influencing anaemia among women aged (15-49 years) -Ghana**

S

| Anaemia occurrence | Difference due to characteristics(E) | | Difference due to coefficient(C) | |
| --- | --- | --- | --- | --- |
|  | Coefficient | Percent (%) | Coefficient | Percent (%) |
| Smoke Cigarette |  |  |  |  |
| No | 1 [reference] | 1[reference] | 1 [reference] | 1[reference] |
| Yes | 0.0010553 | -0.62597 | -0.00068185 | 0.40445 |
| Covered with Health Insurance |  |  |  |  |
| No | 1 [reference] | 1[reference] | 1 [reference] | 1[reference] |
| Yes | 0.0035962 | -2.1332 | 0.0093491 | -5.5456 |
| Current method of contraceptives |  |  |  |  |
| No method |  |  |  |  |
| Folkloric method | 0.0028033 | -1.6628 | -0.003341* | 1.9818 |
| Traditional | 0.000060935 | -0.036145 | -0.0027556 | 1.6345 |
| Modern | -0.0066789 | 3.9617 | -0.0028708 | 1.7028 |
| Reads Newspaper |  |  |  |  |
| No | 1 [reference] | 1[reference] | 1 [reference] | 1[reference] |
| Yes | -0.0060784 | 3.6055 | -0.022909 | 13.589 |
| Watches Television |  |  |  |  |
| No | 1 [reference] | 1[reference] | 1 [reference] | 1[reference] |
| Yes | 0.0038767 | -2.2995 | 0.019823 | -11.758 |
| Listens to Radio |  |  |  |  |
| No | 1 [reference] | 1[reference] | 1 [reference] | 1[reference] |
| Yes | 0.00078494* | 0.969 | 0.001099 | -0.65189 |
| Constant |  |  | -0.050641 | 30.038 |
| Total | -0.00053691 | 0.31848 | -0.16805 | 99.682 |

Supplementary Table *E*: Multivariable decomposition of characteristics influencing anaemia among women aged (15-49years) -Ghana, (Continuation)

Note: P<0.05=*, P<0.01=**, P<0.001=***,

| Anaemia occurrence | Difference due to characteristics (E) | | Difference due to coefficient(C) | |
| --- | --- | --- | --- | --- |
|  | Coefficient | Percent (%) | Coefficient | Percent (%) |
| Age category |  |  |  |  |
| 15-19 |  |  |  |  |
| 20-24 | 0.000024532 | 0.020448 | -0.0016434 | -1.3698 |
| 25-29 | 0.00017388 | 0.14493 | 0.0015427 | 1.2859 |
| 30-34 | 2.8233e-06 | 0.002353 | 0.0084902 | 7.0769 |
| 35-39 | -0.00021674 | -0.18066 | 0.0084544 | 7.0471 |
| 40-44 | 0.00048126 | 0.40115 | -0.0008855 | -0.73809 |
| 45-49 | 0.00039964 | 0.33311 | 0.00050564 | 0.42147 |
| Marital status |  |  |  |  |
| Single | 1 [reference] | 1[reference] | 1 [reference] | 1[reference] |
| Married | -0.00055968 | -0.46651 | 0.071211 | 59.357 |
| Cohabiting | -0.0010257 | -0.85493 | 0.002306 | 1.9222 |
| Separate/Widowed/Divorced | 0.000042202 | 0.035177 | 0.00096602 | 0.80521 |
|  |  |  |  |  |
| Highest level of education |  |  |  |  |
| None | 1 [reference] | 1[reference] | 1 [reference] | 1[reference] |
| Primary | -0.0012424 | -1.0356 | -0.00025301 | -.21089 |
| Secondary | -0.0030626** | -2.5528 | -0.0035704 | -2.976 |
| Higher | -0.00037868 | -.31565 | -0.00018694 | -0.15583 |
|  |  |  |  |  |
| Place of residence |  |  |  |  |
| Urban | 1 [reference] | 1[reference] | 1 [reference] | 1[reference] |
| Rural | -0.00022694 | -0.18916 | 0.013865 | 11.557 |
| Wealth Index |  |  |  |  |
| Poorest | 1 [reference] | 1[reference] | 1 [reference] | 1[reference] |
| Poorer | -0.00015777 | -0.13151 | 0.007197 | 5.999 |
| Mild | -0.00002776 | -0.023139 | 0.0091949 | 7.6643 |
| Richer | -0.00070785* | -0.59001 | -0.0037853 | -3.1552 |
| Richest | 0.00052747*** | 0.43967 | -0.014947 | -12.459 |
| Religion |  |  |  |  |
| Christians | 1 [reference] | 1[reference] | 1 [reference] | 1[reference] |
| Moslems | 0.00093171 | 0.77661 | 0.11072 | 92.293 |
| Traditionalists |  |  |  |  |
| Others | 0.00027488 | 0.22912 | 0.0081032** | 6.7543 |
| Currently working |  |  |  |  |
| No | 1 [reference] | 1[reference] | 1 [reference] | 1[reference] |
| Yes | -0.002684 | -2.2372 | -0.012007 | -10.008 |
| Current pregnancy |  |  |  |  |
| No or not sure | 1 [reference] | 1[reference] | 1 [reference] | 1[reference] |
| Yes | -0.00009628 | -0.080253 | -0.0058785 | -4.8999 |
| Number of children ever born |  |  |  |  |
| 0 | 1 [reference] | 1[reference] | 1 [reference] | 1[reference] |
| 1-3 | 0.00092276 | 0.76915 | -0.030111 | -25.098 |
| 4+ | -0.00008053 | -0.067125 | -0.060281* | -50.247 |
| Currently Breastfeeding |  |  |  |  |
| No | 1 [reference] | 1[reference] | 1 [reference] | 1[reference] |
| Yes | -0.000045558 | -0.037974 | -0.0072346 | -6.0303 |

Supplementary Table F**: Multivariable decomposition of characteristics influencing anaemia among women of reproductive age, Mali.**

Supplementary Table F: Multivariable decomposition of characteristics influencing anaemia among women of reproductive age, Mali (Continuation)

| Anaemia occurrence | Difference due to characteristics(E) | | Difference due to coefficient (C) | |
| --- | --- | --- | --- | --- |
|  | Coefficient | Percent (%) | Coefficient | Percent (%) |
| Smoke Cigarette |  |  |  |  |
| No | 1 [reference] | 1[reference] | 1 [reference] | 1[reference] |
| Yes | 0.00029752 | 0.24799 | 0.00019261 | 0.16055 |
| Covered with Health Insurance |  |  |  |  |
| No | 1 [reference] | 1[reference] | 1 [reference] | 1[reference] |
| Yes | 0.00040812 | 0.34018 | 0.00048696 | 0.40589 |
| Current method of contraceptives |  |  |  |  |
| No method | 1 [reference] | 1[reference] | 1 [reference] | 1[reference] |
| Folkloric method | -0.00036453** | -0.30385 | -0.0016994** | -1.4165 |
| Traditional | -0.00089748** | -0.74808 | -0.00025016 | -0.20851 |
| Modern | -0.0044276** | -3.6906 | 0.00025801 | 0.21506 |
| Reads Newspaper |  |  |  |  |
| No | 1 [reference] | 1[reference] | 1 [reference] | 1[reference] |
| Yes | 0.00031599 | .26339 | .0042042 | 3.5044 |
| Watches Television |  |  |  |  |
| No | 1 [reference] | 1[reference] | 1 [reference] | 1[reference] |
| Yes | -0.0012142 | -1.0121 | -0.00038668 | -0.32231 |
|  |  |  |  |  |
| Listens to Radio |  |  |  |  |
| No | 1 [reference] | 1[reference] | 1 [reference] | 1[reference] |
| Yes | -0.00034085 | -0.28 411 | 0.00060594 | 0.50507 |
| Constant |  |  | 0.027736 | 23.119 |
| Total | -0.012954 | -10.798 | .13293 | 110.8 |

Note: P<0.05=*, P<0.01=**, P<0.001=***

Supplementary Table G**: Multivariable decomposition of characteristics influencing anaemia among women aged (15-49years) -Benin.**

| Anaemia occurrence | Difference due to characteristics (E) | | Difference due to coefficient(C) | |
| --- | --- | --- | --- | --- |
|  | Coefficient | Percent (%) | Coefficient | Percent (%) |
| Age category |  |  |  |  |
| 15-19 | 1 [reference] | 1[reference] | 1 [reference] | 1[reference] |
| 20-24 | -0.00051298 | -.31402 | -0.0005058 | -.30963 |
| 25-29 | 0.0012132** | 0.47147 | -0.012287 | -7.5216 |
| 30-34 | 0.00077018 | 0.74268 | -0.0033817 | -2.0701 |
| 35-39 | 0.0005696 | 0.34868 | 0.00030389 | 0.18603 |
| 40-44 | -0.00029466 | -0.18038 | 0.0015508 | 0.94935 |
| 45-49 | -0.00058525 | -0.35826 | -0.0022206 | -1.3593 |
|  |  |  |  |  |
| Marital status |  |  |  |  |
| Single | 1 [reference] | 1[reference] | 1 [reference] | 1[reference] |
| Married | -0.00015144 | -0.092705 | -0.024823 | -15.195 |
| Cohabiting | -0.0013703 | -0.83882 | -0.014687 | -8.9908 |
| Separate/Widowed/Divorced | 0.00012335 | 0.075509 | -0.0025531 | -1.5629 |
|  |  |  |  |  |
| Highest level of education |  |  |  |  |
| No Education | 1 [reference] | 1[reference] | 1 [reference] | 1[reference] |
| Primary | 0.000010998 | 0.006732 | -0.0015602 | -0.95507 |
| Secondary | -0.00092863 | -0.56846 | -0.0017866 | -1.0937 |
| Higher | -0.000038695 | -0.023687 | -0.00032044 | -0.19616 |
|  |  |  |  |  |
| Place of residence |  |  |  |  |
| Urban | 1 [reference] | 1[reference] | 1 [reference] | 1[reference] |
| Rural | -0.00074661 | -0.45704 | -0.0052676 | -3.2246 |
|  |  |  |  |  |
| Wealth Index |  |  |  |  |
| Poorest | 1 [reference] | 1[reference] | 1 [reference] | 1[reference] |
| Poorer | -0.00012183* | -0.074576 | -0.0056374 | -3.451 |
| Mild | -0.00059936* | -0.3669 | -0.0094692 | -5.7966 |
| Richer | -0.0011795*** | -0.72202 | -0.0048221 | -2.9519 |
| Richest | 0.0019855** | 1.2154 | -0.016862 | -10.322 |
|  |  |  |  |  |
| Religion |  |  |  |  |
| Christians | 1 [reference] | 1[reference] | 1 [reference] | 1[reference] |
| Moslems | -0.0010015 | -0.61305 | 0.001249 | 0.76458 |
| Traditionalists | 0.00026083 | 0.15967 | 0.0011808 | 0.72284 |
| Others | -0.00017472 | -0.10696 | 0.0017918 | 1.0968 |
|  |  |  |  |  |
| Currently working |  |  |  |  |
| No | 1 [reference] | 1[reference] | 1 [reference] | 1[reference] |
| Yes | -0.0032058 | -1.9624 | -0.03251* | -19.901 |
| Current pregnancy |  |  |  |  |
| No or not sure | 1 [reference] | 1[reference] | 1 [reference] | 1[reference] |
| Yes | 0.0016445*** | 1.0067 | 0.011626** | 7.1171 |
|  |  |  |  |  |
| Number of children ever born |  |  |  |  |
| 0 | 1 [reference] | 1[reference] | 1 [reference] | 1[reference] |
| 1-3 | -0.00090588 | -0.55454 | 0.039761* | 24.34 |
| 4+ | 0.00042742 | 0.26164 | 0.042255* | 25.866 |

Supplementary Table G: Multivariable decomposition of characteristics influencing anaemia among women aged (15-49years) -Benin (Continuation)

| Anaemia occurrence | Difference due to characteristics (E) | | Difference due to coefficient(C) | |
| --- | --- | --- | --- | --- |
|  | Coefficient | Percent (%) | Coefficient | Percent (%) |
| Currently Breastfeeding |  |  |  |  |
| No | 1 [reference] | 1[reference] | 1 [reference] | 1[reference] |
| Yes | 0.000025974 | 0.0159 | -0.0071519 | -4.3781 |
| Smoke Cigarette |  |  |  |  |
| No | 1 [reference] | 1[reference] | 1 [reference] | 1[reference] |
| Yes | 0.00098708 | 0.60425 | 0.000055055 | 0.033702 |
| Covered with Health Insurance |  |  |  |  |
| No | 1 [reference] | 1[reference] | 1 [reference] | 1[reference] |
| Yes | 0.00067065 | 0.41054 | -0.0012929 | -0.79148 |
| Current method of contraceptives |  |  |  |  |
| No method |  |  |  |  |
| Folkloric method |  |  |  |  |
| Traditional |  |  |  |  |
| Modern |  |  |  |  |
| Reads Newspaper |  |  |  |  |
| No | 1 [reference] | 1[reference] | 1 [reference] | 1[reference] |
| Yes | 0.00057037 | 0.34915 | 0.0003917 | 0.23978 |
| Watches Television |  |  |  |  |
| No | 1 [reference] | 1[reference] | 1 [reference] | 1[reference] |
| Yes | 0.000094096 | 0.057601 | 0.011177 | 6.8418 |
| Listens to Radio |  |  |  |  |
| No | 1 [reference] | 1[reference] | 1 [reference] | 1[reference] |
| Yes | -0.0022045* | -1.3495 | -0.023915** | -14.64 |
| Constant |  |  | 0.22774*** | 139.41 |
| Total | -0.00099946 | -0.61182 | 0.16436 | 100.61 |

Note: P<0.05=*, P<0.01=**, P<0.001=***,

Supplementary Table H**: Multivariable decomposition of characteristics influencing anaemia among women aged (15-49years) Sierra Leone**

| Anaemia occurrence | Difference due to characteristics (E) | | Difference due to coefficient (C) | |
| --- | --- | --- | --- | --- |
|  | Coefficient | Percent (%) | Coefficient | Percent (%) |
| Age category |  |  |  |  |
| 15-19 | 1 [reference] | 1[reference] | 1 [reference] | 1[reference] |
| 20-24 | -0.00053546*** | 10.856 | -0.0074904 | 151.86 |
| 25-29 | 0.0062247*** | -126.2 | -0.04018 | 814.62 |
| 30-34 | -0.001156*** | 23.438 | -0.031042 | 629.36 |
| 35-39 | 0.0033912*** | -68.755 | -0.042997 | 871.74 |
| 40-44 | 0.0017843*** | -36.175 | -0.024781 | 502.43 |
| 45-49 | -0.0017484*** | 35.447 | -0.017647 | 357.78 |
|  |  |  |  |  |
| Marital status |  |  |  |  |
| Single | 1 [reference] | 1[reference] | 1 [reference] | 1[reference] |
| Married | -0.0012413 | 5.168 | 0.09812 | -1989.3 |
| Cohabiting | 0.00030034 | 0.0893 | 0.0039341 | -79. 762 |
| Separate/Widowed/Divorced | 0.00029387** | 0.9581 | 0.020034 | -406.18 |
|  |  |  |  |  |
| Highest level of education |  |  |  |  |
| No Education | 1 [reference] | 1[reference] | 1 [reference] | 1[reference] |
| Primary | -0.000021045 | 0.42669 | 0.0041518 | -84.176 |
| Secondary | -0.0020876 | 42.325 | -0.008062 | 163.45 |
| Higher | -0.00081683 | 16.561 | 0.00079219 | -16.061 |
|  |  |  |  |  |
| Place of residence |  |  |  |  |
| Urban | 1 [reference] | 1[reference] | 1 [reference] | 1[reference] |
| Rural | -0.0010414* | 21.114 | 0.11504 | -2332.4 |
| Wealth Index |  |  |  |  |
| Poorest | 1 [reference] | 1[reference] | 1 [reference] | 1[reference] |
| Poorer | 0.00014315 | -2.9023 | -0.014263 | 289.17 |
| Mild | 0.00012918 | -2.6191 | -0.006528 | 132.35 |
| Richer | 0.00015803 | -3.2041 | 0.0042122 | -85.399 |
| Richest | -0.0025744** | 52.195 | -0.020984 | 425.44 |
|  |  |  |  |  |
| Religion |  |  |  |  |
| Christians | 1 [reference] | 1[reference] | 1 [reference] | 1[reference] |
| Moslems | 0.0016545*** | -33.545 | .0074084 | -1502 |
| Traditionalists |  |  |  |  |
| Others | -0.00072234 | 14.645 | 0.0030332 | -61.496 |
|  |  |  |  |  |
| Currently working |  |  |  |  |
| No | 1 [reference] | 1[reference] | 1 [reference] | 1[reference] |
| Yes | 0.000075358 | -1.5279 | -0.01868 | 378.73 |
| Current pregnancy |  |  |  |  |
| No or not sure | 1 [reference] | 1[reference] | 1 [reference] | 1[reference] |
| Yes | -2.8105e-06** | 0.056982 | -0.012641 | 256.29 |
|  |  |  |  |  |
| Number of children ever born |  |  |  |  |
| 0 | 1 [reference] | 1[reference] | 1 [reference] | 1[reference] |
| 1-3 | -0.0011271 | 22.851 | 0.0055599 | -112.72 |
| 4+ | -0.00029839 | 6.0497 | 0.044736 | -906.99 |
| Currently Breastfeeding |  |  |  |  |
| No | 1 [reference] | 1[reference] | 1 [reference] | 1[reference] |
| Yes | -0.00083679 | 16.966 | -0.0039069 | 79.21 |

Supplementary Table H: Multivariable decomposition of characteristics influencing anaemia among women aged (15-49years) Sierra Leone (Continuation)

| Anaemia occurrence | Difference due to characteristics (E) | | Difference due to coefficient(C) | |
| --- | --- | --- | --- | --- |
|  | Coefficient | Percent (%) | Coefficient | Percent (%) |
| Smoke Cigarette |  |  |  |  |
| No | 1 [reference] | 1[reference] | 1 [reference] | 1[reference] |
| Yes | 0.00029091 | -5.898 | 0.0050513 | -102.41 |
|  |  |  |  |  |
| Covered with Health Insurance |  |  |  |  |
| No | 1 [reference] | 1[reference] | 1 [reference] | 1[reference] |
| Yes | -0.00063727 | 12.92 | 0.0075822 | -153.73 |
| Current method of contraceptives |  |  |  |  |
| No method | 1 [reference] | 1[reference] | 1 [reference] | 1[reference] |
| Folkloric method | -0.00048479 | 9.8289 | 0.0014405 | -29.205 |
| Traditional | 0.00046244 | -9.3757 | -0.00358 | 72.584 |
| Modern | -0.0076938*** | 155.99 | -0.0030635 | 62.112 |
| Reads Newspaper |  |  |  |  |
| No | 1 [reference] | 1[reference] | 1 [reference] | 1[reference] |
| Yes | 0.00004877 | -0.98879 | 0.0080737 | -163.69 |
| Watches Television |  |  |  |  |
| No | 1 [reference] | 1[reference] | 1 [reference] | 1[reference] |
| Yes | 0.00075179 | -15.242 | 0.0056882 | -115.33 |
|  |  |  |  |  |
| Listens to Radio |  |  |  |  |
| No | 1 [reference] | 1[reference] | 1 [reference] | 1[reference] |
| Yes | -0.0017806 | 6.101 | -0.0047616 | 96.54 |
| Constant |  |  | -0.13673 | 2772.1 |
| Total | -.0091288 | 185.08 | .0041965 | -85.083 |

Note: P<0.05=*, P<0.01=**, P<0.001=***,
